# Computational design of low-volatility lubricants for space using interpretable machine learning

**DOI:** 10.1186/s13321-026-01250-1

**Published:** 2026-07-02

**Authors:** Daniel Miliate, Ashlie Martini

**Affiliations:** Department of Mechanical and Aerospace Engineering, University of California Merced, Merced, CA 95343 USA

## Abstract

**Abstract:**

The function and lifetime of moving mechanical assemblies (MMAs) in space depend on the properties of lubricants. MMAs that experience high speeds or high cycles require liquid-based lubricants due to their ability to reflow to the point of contact. However, only a few liquid-based lubricants have vapor pressures low enough for the vacuum conditions of space, each of which has limitations that add constraints to MMA designs. This work introduces a data-driven machine learning (ML) approach to predicting vapor pressure, enabling virtual screening and discovery of new space-suitable liquid lubricants. The ML models are trained with data from both high-throughput molecular dynamics simulations and experimental databases. The models are designed to prioritize interpretability, enabling the relationships between chemical structure and vapor pressure to be identified. Based on these insights, several candidate molecules are proposed that may have promise for future space lubricant applications in MMAs.

**Scientific contribution:**

This work develops interpretable machine learning models for vapor pressure of low volatility molecules, identifying the structural features that drive volatility. The framework is accurate in the ultra-low-volatility regime where existing methods are unreliable, enabling virtual screening of candidate space lubricants. All code and data are publicly available, and the approach generalizes to materials discovery in other extreme environments.

**Graphical abstract:**

**Figure:**
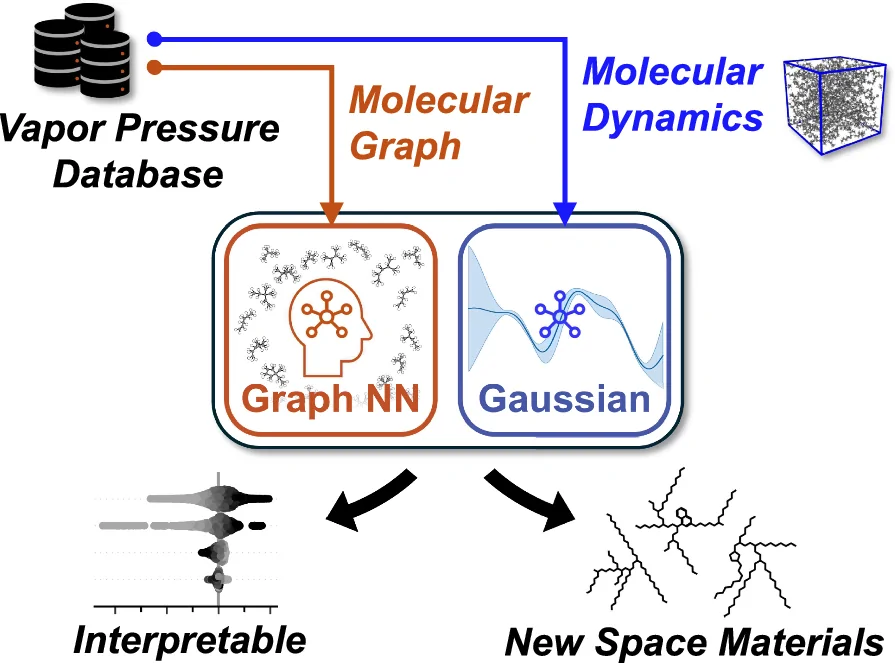

## Introduction

Moving mechanical assemblies (MMAs) in spacecraft, such as gimbals, actuators, and reaction wheels, require reliable lubrication to reduce friction and minimize wear [1–3]. On Earth, this requirement is commonly met using liquid-based lubricants like oils or greases. In space, however, the performance of lubricants is constrained by exposure to vacuum, radiation, and wide operating temperatures. Only a narrow subset of liquid-based lubricants can function in such conditions; common space lubricants include polyalphaolefins (PAOs), perfluoropolyethers (PFPEs), and multiply-alkylated cyclopentanes (MACs) [1].

Within this select family of lubricant chemistries, trade-offs must still be made to balance additive compatibility [4–7], recommended operating temperatures [8], and evaporative risk [9]. PFPEs are incompatible with many additive packages that improve wear resistance [4, 6], whereas MACs and PAOs can incorporate such additives [5–7]. Another limitation of PFPEs is that they are per- and polyfluoroalkyl substances whose use is increasingly limited by regulation. At extremely low temperatures, the viscosity increase exhibited by all three chemistries often require heaters, which add mass and power demands [1, 10]. PFPEs can operate at slightly lower temperatures than MACs and PAOs because the rate of change of viscosity with temperature is slower [11, 12], eliminating the need for heaters in some cases. All liquid-based lubricants risk evaporation when in vacuum, but MACs and PFPEs are preferred over PAOs in vacuum exposed conditions because of their lower vapor pressures [13]. These tradeoffs constrain overall design flexibility, affecting mission cost and capability. Expanding the pool of space-suitable liquid-based lubricants could alleviate these limitations.

Among the various properties of space lubricants, vapor pressure is one of the most critical to assess early in the qualifying processing, since even slow evaporation can lead to lubricant supply loss or contamination of sensitive components [14–16]. Methods for determining vapor pressure generally fall into three categories: direct experimental measurement with semi-empirical models, molecular modeling, and machine learning.

Experimental methods for measuring vapor pressure [17], such as thermogravimetric analyzer [18] or Knudsen effusion [19, 20], are based on evaporation in vacuum. Data measured using these instruments can be fit to semi-empirical models, like the Clausius-Clapeyron [21–23] and Antoine’s equations [24, 25], to enable vapor pressure to be estimated using interpolation. However, the empirical constants are only valid for the materials and temperature ranges of the original measurements. This limits their applicability to novel or untested molecules, leaving a gap in the ability to rapidly evaluate vapor pressures of new substances or expanded temperature ranges for heritage lubricants.

Molecular modeling has been used to estimate vapor pressure using either Monte Carlo (MC) [26–29] or Molecular Dynamics (MD) [26, 30–32] simulations. In principle, these simulations model both vapor and liquid phase molecules in equilibrium, or vapor-liquid equilibrium (VLE). In VLE, the chemical potentials of the two phases are equal, and the vapor pressure can be determined by applying the virial theorem [33, 34] to the molecules in the vapor phase [29]. In addition to VLE, vapor pressure can be determined by simulating the pure vapor phase [30]. The vapor pressure of volatile liquids, either in VLE or pure vapor phase, can be performed using simulations with only a few thousands of atoms such that the simulations can be run with minimal computational costs and runtimes. For larger, low-volatility molecules, however, phase transitions occur too infrequently to practically achieve VLE, often requiring specialized modeling strategies [31]. In addition to low-volatility limitation, the accuracy of MD simulations depends heavily on the accuracy of the force field [32]. For novel molecular structures, especially those outside the chemical space for which force fields have been validated, simulation predictions can be unreliable. Thus, molecular simulations remain limited in their practical application to low-volatility molecules, as they are both computationally demanding and sensitive to force field accuracy. These challenges in molecular simulations create a need for alternative approaches that can rapidly and accurately estimate vapor pressure without extensive parameterization.

Some of the limitations of experiments and molecular simulations are overcome with machine learning (ML) models. Early ML models of vapor pressure used architectures such as multiple linear regression and simple neural networks [35–40]. These ML models relied on 2D molecular descriptors, or features, calculated from quantum chemistry. However, 2D molecular features cannot necessarily capture intermolecular interactions. More robust features derived from MD simulations have been used in Gaussian Process Regression (GPR) models with success but have not been applied to vapor pressure [41]. In recent years, graph neural networks (GNN) have been used to predict vapor pressure [42, 43] and related properties like boiling point [44–46] from graph representations of molecules. In other applications, GNNs have offered an avenue to explain which structural factors contributed to the predicted property by determining the attribution of substructures [47]. However, current vapor pressure GNNs have not implemented these substructure masking techniques which inhibits interpretation of structure–property relationships. Additionally, most existing ML models have been trained on datasets dominated by relatively volatile compounds, making them less useful for space-tribology applications where volatile compounds are rarely used, with the exception of highly specific use cases (e.g., gas bearings in Stirling converters [48]). These factors highlight a need for ML models that are both trained from space-lubricant-relevant datasets and capable of explaining their predictions.

In this work, interpretable ML models for space lubricant discovery were developed using GPR and GNN architectures tailored for predicting low vapor pressure. The GPR model was trained on a curated dataset combining experimental vapor pressure values with both static molecular descriptors (e.g., molecular weight) and dynamic molecular descriptors (e.g., radius of gyration) from short equilibrium MD simulations. These descriptors provided single-point representations of each molecule and were incorporated as features in the GPR models. The GPR approach was complemented by GNN models that leveraged molecular graph representations, where atoms served as nodes and chemical bonds as edges. The graph representations were processed through a relational graph convolutional network, allowing the model not only to learn structural patterns (e.g., aromatic substructures or functional groups), and attribution methods can subsequently be applied to analyze the contributions of these substructures. Screening a large set of virtually generated molecular structures with this framework yielded several novel structures with predicted vapor pressures comparable to those of established space lubricants. Altogether, this work demonstrates the value of interpretable ML in advancing vapor pressure predictability as the first step toward space lubricant discovery while establishing transferable design principles that can guide the development of future space-qualified materials.

## Methods

### Target data

**Fig. 1:**
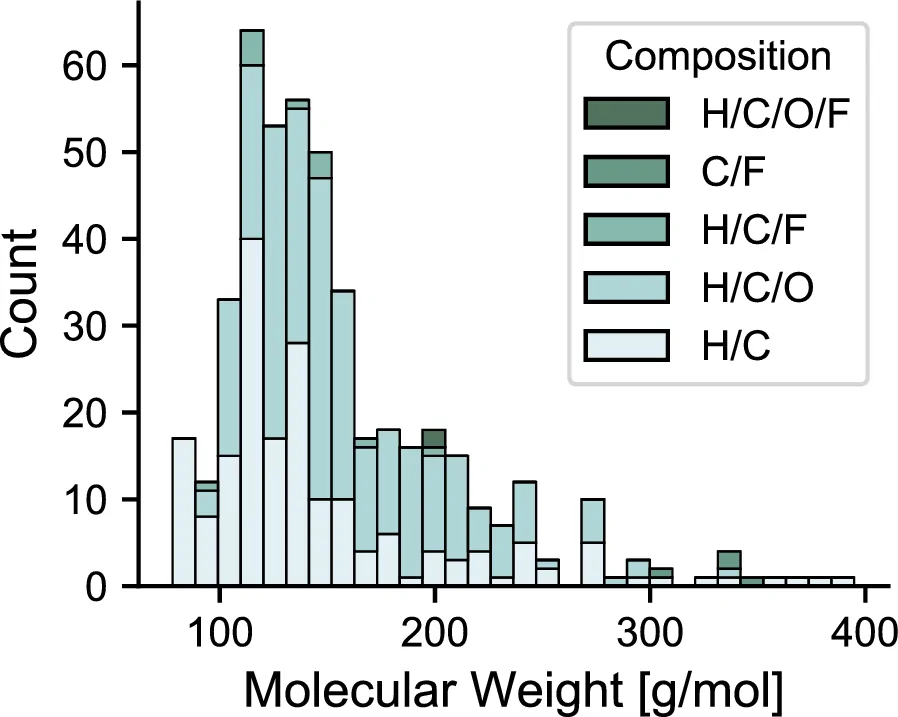
Stacked distribution of molecular weights for H/C, H/C/O, H/C/F, C/F, and H/C/O/F elemental compositions

Molecular data were obtained from the NIST Chemistry WebBook [49], which included InChIKeys and Antoine’s equation parameters. To ensure relevance to the range of chemistries in space-qualified lubricants, the dataset was restricted to molecules composed of carbon, hydrogen, oxygen, and fluorine. These elements were combined in patterns representative of PFPE-, MAC-, and PAO-like chemistries (H/C, H/C/O, H/C/F, C/F, and H/C/O/F). A minimum of six carbon atoms per molecule was required to exclude small, highly volatile compounds unlikely to meet operational constraints for space lubrication. Molecules differing only by geometric isomerism were consolidated by retaining the more energetically stable form. This calculation was performed through geometry optimization of each isomer at the B3LYP [50–52]/6-31 G* [53] level of theory using Gaussian 16 Rev. B.01 [54]. Deuterated species were removed. After these steps, 460 unique molecules from NIST remained. The number of molecules having H/C, H/C/O, H/C/F, C/F, and H/C/O/F chemistries were 188, 255, 11, 4, and 2, respectively. The distribution of molecular weights is visualized in Fig. 1, where contributions from each chemistry are stacked and color-coded. Note that data was available for relatively few fluorinated systems, so the models were primarily trained on hydrocarbons.

The target data, vapor pressure, was calculated from Antoine’s equation parameters reported in the NIST Chemistry WebBook. Multiple Antoine parameter sets were available with overlapping validated temperature ranges for 34 molecules. For each of these molecules, vapor pressures calculated within the shared temperature interval were compared. For 30 molecules, the mean absolute difference between the Antoine-calculated vapor pressures in $\log _{10}$ was less than 0.05 Pa. For the other four molecules, the mean absolute difference was less than 0.15 Pa. This suggests that there is generally good agreement where overlapping temperatures exists. Despite this consistency, systematic uncertainties inherent to the original experimental measurements may persist and represent an unavoidable limitation of dataset. This is particularly relevant for low-volatility substances, where impurities, e.g., residual reactants, and limitations in temperature control can contribute to measurement uncertainty. In cases where multiple sets of Antoine’s parameters were reported, we used those fit to the widest temperature range.

In addition to the NIST data, vapor pressure data for MACs was determined directly from previously reported experimental measurements [6, 18, 55–58]; see Figure S1. Note that Ref. [57] reported two values for 1,3-bis(2-octyldodecyl) that were orders of magnitude higher than those in the other references. These were identified as outliers and not included in subsequent analyses. The MAC vapor pressure data was fit to the Antoine equation such that that equation could be used to calculate vapor pressure at temperatures between the experimentally measured data points. For 1,3,4-tri-(2-octyldodecyl) cyclopentane, the R^2^ and root mean squared error (RMSE) of the Antoine fit were 0.967 and 0.597 Pa, respectively. For bis(2-octyldodecyl) cyclopentane, the R^2^ and RMSE were 0.985 and 0.318 Pa, respectively.

Since the validated temperature ranges were not standardized, a single temperature could not be applied to all compounds. Instead, two datasets and featurization approaches were adopted. In the first approach, vapor pressures were calculated at 20 evenly spaced temperatures between the minimum and maximum temperatures in the validated range. The sampled temperatures were separated by at least 2 K to limit correlation between instances and remain within the practical temperature control limits of the MD simulations. The dataset included vapor pressures at temperatures from 178 K to 723 K, with a median of 401 K; see Figure S2. This resulted in a variable-temperature dataset containing 462 unique molecular structures and a total of 9,240 vapor pressure instances. In the second approach, a fixed-temperature dataset was created. Vapor pressure was calculated for 337 molecules at 387 K, the temperature that maximized the number of molecules at a single temperature within their validated range.

In both approaches, vapor pressure was $\log _{10}$-transformed to balance loss contributions across many orders of magnitudes. Featurization of the two target datasets differed, being tailored to either GPR or GNN model architectures. Although other ML architectures such as random forests or gradient boosting machines may provide competitive performance, GPR and GNN were chosen because of the emphasis of this study on interpretability given the size of the dataset. The GPR and GNN model architectures were developed to complement each other. The GPR model uses MD derived descriptors, grounding the inputs to a physical model and enabling predictions as a function of temperature. However, it does not directly provide molecular structure–property relationship. This is instead satisfied by the GNN model, which directly correlates structures and properties at a fixed temperature, 387 K.

### Gaussian process regression models

Featurization for GPR models was designed to capture both static and dynamic molecular behavior, utilizing the variable-temperature dataset. Static descriptors, such as molecular weight and elemental counts, were calculated from SMILES strings [59] using RDKit [60]. Dynamic descriptors, such as radius of gyration, were obtained from short non-reactive equilibrium MD simulations using PyL3dMD [61], instead of simulating vapor pressure directly. The MD workflow and simulation steps are detailed in the Supplementary Information (SI). The temperature used in the simulation corresponded to the temperature of the vapor pressure calculation in the dataset. The dynamic descriptors from the MD simulations vary at each sampled timestep. So, the PyL3dMD package was modified to output aggregate statistics: the average, standard deviation, minimum, 25th percentile, 50th percentile, 75th percentile, and maximum feature value for all molecules across all sampled frames. Analysis of the time variation of the dynamic descriptors confirmed that the sampling time was sufficient and there was little variation after the simulation reached steady-state; see Figure S3. This process produced more than 14,000 features, derived from dynamic and static molecular descriptors.

The large number of features required dimensionality reduction to improve the computational efficiency of model development. Three key techniques were applied. First, any feature with the same value for every molecule was removed. Second, redundant features were filtered by examining pairwise correlations; when two features had an R^2^ greater than 0.5, one was discarded. These two filters reduced the dataset to 35 static and 426 dynamic features. The third technique was performed using the Least Absolute Shrinkage and Selection Operator (LASSO) [41, 62, 63]. LASSO was chosen because it reduces model complexity by shrinking the coefficients of less influential features toward zero, effectively retaining only the most predictive variables.

To ensure robust evaluation of the remaining features with LASSO, the dataset was split into four partitions. The variable-temperature dataset contained multiple instances of the same molecular structure. However, molecules were kept to a single partition to avoid data leakage, i.e., not spread across multiple partitions. Stratification was applied to maintain a balanced distribution of target values across partitions. Because multiple vapor pressure values were available per molecule, the median was used for stratification. Three partitions were used for outer three-fold cross-validation (CV), and one was reserved as a holdout test set. Figure S4 and Figure S5 show the stratified data split and cross validation workflow, respectively.

**Fig. 2:**
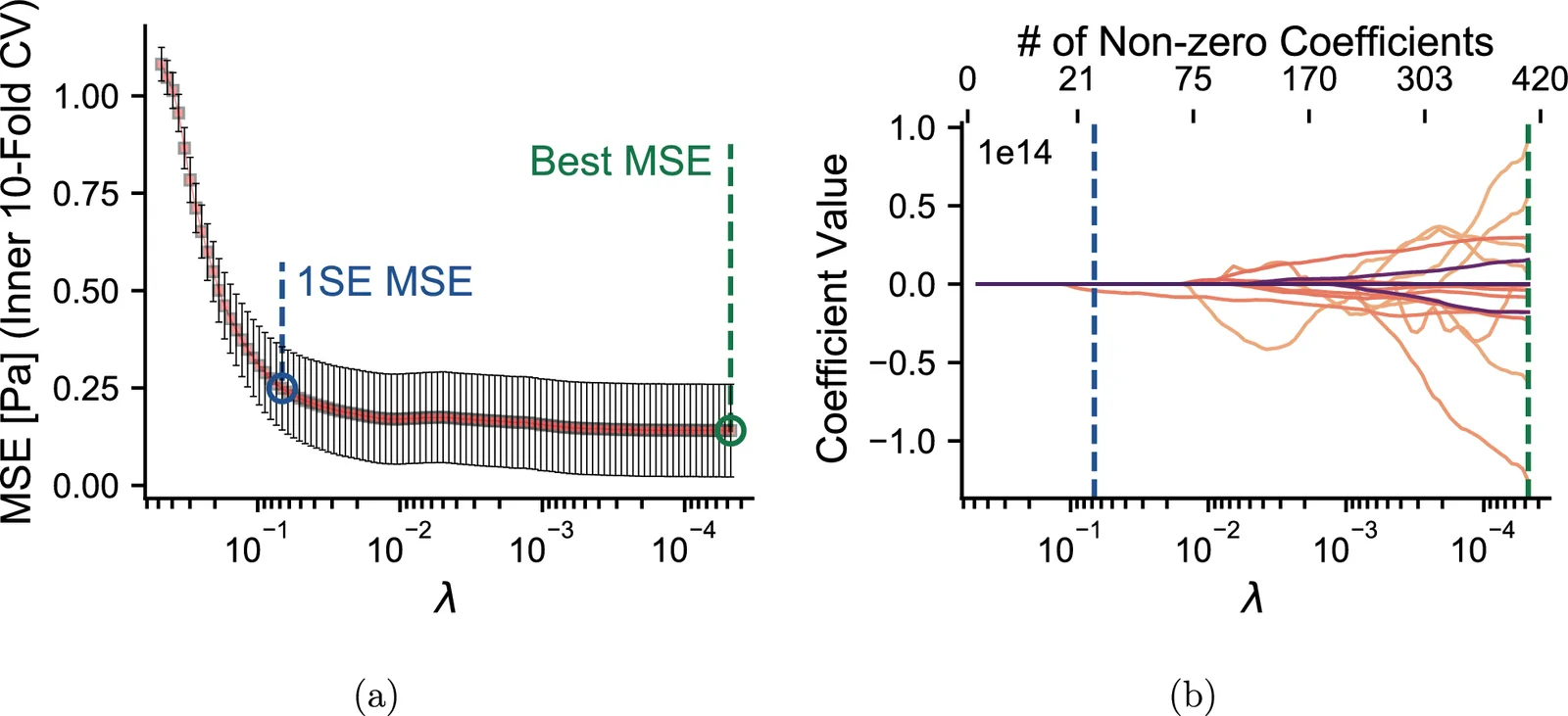
Representative **a** MSE vs λ hyperparameter and **b** coefficient vs λ hyperparameter from LASSO. In (**a**), the error bars are calculated from the inner 10-fold CV. The green dashed vertical line indicates the λ hyperparameter value that resulted in the best (minimum) mean MSE. The dashed blue vertical line is one standard error (1SE) greater than the best MSE. In (**b**), feature coefficients are shown as a function of the λ hyperparameter, with the corresponding number of non-zero coefficients on the top axis

Three LASSO models were trained using the three outer CV fold partitions. In each LASSO model, the two training folds were combined, and an inner 10-fold CV with random splits was implemented. The regularization parameter, λ, can be varied to decrease the coefficient of less predictive features. The final λ value was determined using the one-standard-error (1SE) rule, balancing model accuracy and simplicity. This rule selected the λ value corresponding with the mean squared error (MSE) of the 10-fold CV within one standard deviation of the minimum MSE observed [41]. Figure 2a and b illustrate the λ optimization curve and corresponding coefficient traces. Features with non-zero coefficients in the three 1SE models were combined, resulting in a final set of 51 unique features (8 static and 43 dynamic).

GPR models were trained with the variable-temperature dataset using systematically constructed feature subsets [41]. From the 51 available features, all unique one-, two-, three-, and four-feature combinations were evaluated, yielding 51, 1,275, 20,825 and 249,900 GPR models, respectively.

The initial training of feature-subset GPR models was performed with three-fold cross-validation to determine the best feature subsets, using *constant* and *matern52* and the basis and kernel functions. Data was standardized for each feature subset using the training set for each cross-validation. All models with an average R^2^ > 0.7 were retrained and cross validated with all combinations of the basis functions, including *none*, *linear*, and *pureQuadratic*, and kernel functions, including *exponential*, *squaredexponential*, *matern32*, *matern52*, *rationalquadratic*, *ardexponential*, *ardsquaredexponential*, *ardmatern32*, *ardmatern52*, and *ardrationalquadratic*. The best basis and kernel function combination was recorded for each feature set. Models with average R^2^ > 0.85 were retrained for the final evaluation. The three CV folds were used as the training set and benchmarked against the holdout test set. Models with a final evaluation R^2^ > 0.9 were kept and retrained using all the data available. Although models were filtered using R^2^, additional metrics are listed for completeness, such as RMSE, mean absolute error (MAE), and mean absolute percent error (MAPE). Both the LASSO and GPR models were trained using MATLAB [64].

### Graph neural network models

The GNN architecture and featurization used in this study followed one used to successfully predict aqueous solubility, mutagenicity, hERG-related cardiotoxicity and blood-brain barrier permeation [47]. The molecules from the fixed-temperature datasets were represented as molecular graphs, as opposed to static and dynamic molecular descriptors. Further discussion can be found in the original work [47], but the GNN architecture is a relational graph convolution network (RCGN). The RCGN used the molecular graph representation as feature inputs with masking, i.e., graph nodes as atoms, functional groups, BRICS [65] or Murcko substructures [66, 67]. The masking allowed for the determination of attribution to the prediction for atoms and substructures.

To train the RCGN, hyperparameters were explored first, and are available in Table 1. The fixed-temperature dataset was limited in size and instead used an 80:10:10 split for training, validation and holdout test splits. The best set of hyperparameters was then used to train 10 RCGN models with different weighting seeds. The objective function used during training was the mean squared error.

**Table 1:** Hyperparameter optimization for RCGN model

RCGN Hidden Layers (2-layer)	[32, 32], [64, 64], [128, 128], [256, 256]
RCGN Hidden Layers (3-layer)	[32, 32, 32], [64, 64, 64], [128, 128, 128], [256, 256, 256]
FFN Hidden Features	[32, 64, 128, 256]
FFN Drop Out	[0, 0.1, 0.2, 0.3, 0.4, 0.5, 0.6, 0.7, 0.8]
RGCN Drop Out	[0, 0.1, 0.2, 0.3, 0.4, 0.5, 0.6, 0.7, 0.8]
Learning Rate	[0.05, 0.01, 0.003, 0.001, 0.0003, 0.0001, 0.00001]

The computational framework developed here is available on Github (see Computational Availability Statement) such that it can be used or adapted in future work. Particularly, the Github contains the training datasets, the GNN and GPR models with three representative molecules as a demonstration of how to use the trained models, and a sample LAMMPS script with expected outcomes.

## Results and discussion

### GPR and GNN model interpretation

GPR models with only one and two features exhibited poor predictive performance. Introducing a third feature markedly improved the model accuracy, yielding one model with an R^2^ > 0.9 on the holdout test set. Of the four-feature models, 34 had an R^2^ > 0.9 for the holdout test set. Details of the top-performing three- and four-feature models, including their features and evaluation metrics, are summarized in Table 2. A summary of the performance metrics for all 34 four-features GPR models is available in Table S1.

**Fig. 3:**
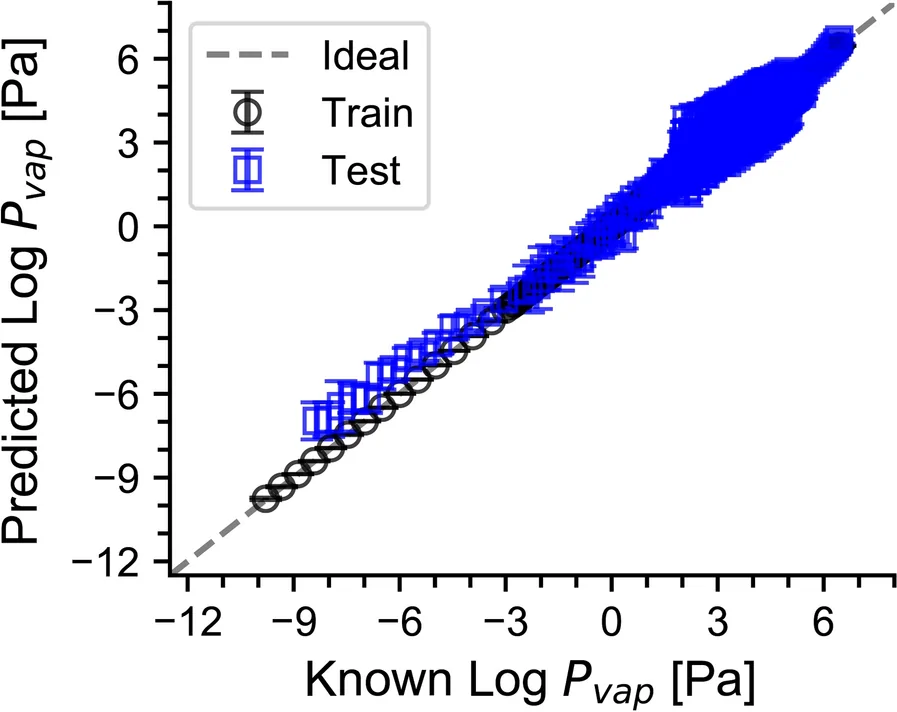
Parity plot of log vapor pressure ($P_{vap}$) of the temperature-dependent dataset containing 20 instances per molecule. The symbols and error bars represent the mean and standard deviation as predicted by the ten GPR models in the final evaluation

From the 34 four-feature models, the ten best models were selected to form an ensemble GPR model, designed to capture complementary predictive strengths and reduce individual model bias. The average prediction and associated uncertainties of the final evaluation are shown in Fig. 3; deviation plot in Figure S6. The ensemble model demonstrated strong predictive performance, i.e., better than any individual model, with R^2^ of 0.946, RMSE of 0.296 Pa, MAE of 0.201 Pa, and MAPE of 6.44% for the holdout test set. The ability of the model to reproduce thermodynamic consistency was also verified by confirming that the holdout test set exhibited linearity in log vapor pressure vs. inverse temperature; see Figure S7.

In addition to overall predictive performance, it is important that the GPR ensemble model predicts very low vapor pressure instances accurately. The 1,3-bis(2-octyldodecyl) cyclopentane molecule, which has the lowest vapor pressure instances in the test set, was predicted with R^2^ of 0.939, RMSE of 0.646 Pa, MAE of 0.573 Pa, and MAPE of 27.9%. The R^2^, RMSE, and MAE signify that the model is still within one order of magnitude accuracy in predicting vapor pressure of the 1,3-bis(2-octyldodecyl) cyclopentane molecule. As a benchmark, a comparison between our GPR model, Chemprop VP [68], and GRAPPA [69] is available in Figure S8. Our GPR model provides the most accurate predictions at low vapor pressures that are relevant for space lubricants. Following the final evaluation, all GPR models in the ensemble were retrained using the complete dataset to improve robustness and to examine relationships between molecular features and predicted vapor pressure.

**Table 2:** Top three- and four-feature subset GPR models performance metrics in the final evaluation

Features Names	Training Split				Test Split			
	R^2^	RMSE (Pa)	MAE (Pa)	MAPE (%)	R^2^	RMSE (Pa)	MAE (Pa)	MAPE (%)
*Temperature*	0.980	0.162	0.109	3.39	0.907	0.389	0.249	8.37
$${Density}_{mean}$$								
$${getawayHATSIP2}_{min}$$								
*Temperature*	0.999	0.001	0.0004	0.014	0.940	0.312	0.231	7.41
$${Density}_{mean}$$								
$${getawayHATSIP2}_{min}$$								
$${FPSA3}_{mean}$$								
*Temperature*	0.999	0.001	0.001	0.017	0.930	0.338	0.236	8.59
$${Density}_{mean}$$								
$${getawayHATSIP2}_{min}$$								
$${MoRSEu23}_{max}$$								
*Temperature*	0.998	0.056	0.038	1.26	0.925	0.348	0.239	8.47
$${Density}_{mean}$$								
$${getawayHATSIP2}_{min}$$								
$${Density}_{std}$$								

Only one three-feature model had an R^2^ greater than 0.9. The top four-features models contained the same three features, indicating high predictive ability

**Fig. 4:**
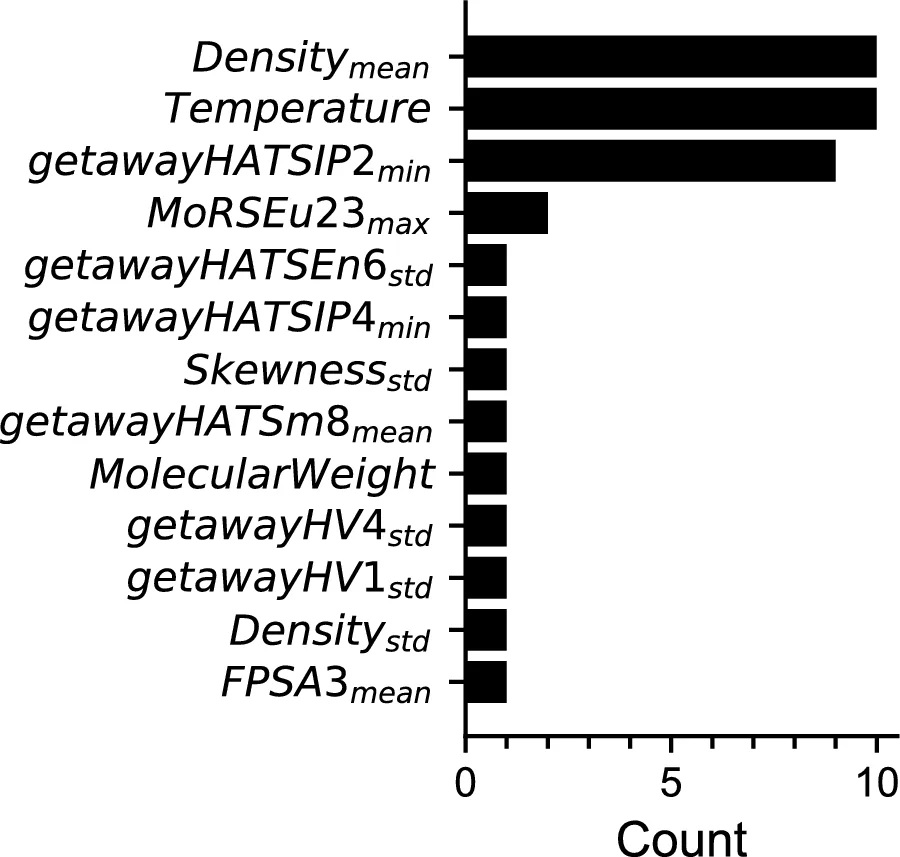
Histogram of all features in the top ten four-feature GPR models. *Temperature*, ${getawayHATSIP2}_{min}$, and ${Density}_{mean}$ appear in nearly all GPR models

A histogram of the features in the top ten four-feature GPR models is shown in Fig. 4. Nine of the top ten four-feature models shared the same three features: *Temperature*, ${getawayHATSIP2}_{min}$, and ${Density}_{mean}$. The best-performing four-feature model, incorporating *Temperature*, ${getawayHATSIP2}_{min}$, ${Density}_{mean}$, and ${FPSA3}_{mean}$, was selected as a representative model for interpretability analysis.

**Fig. 5:**
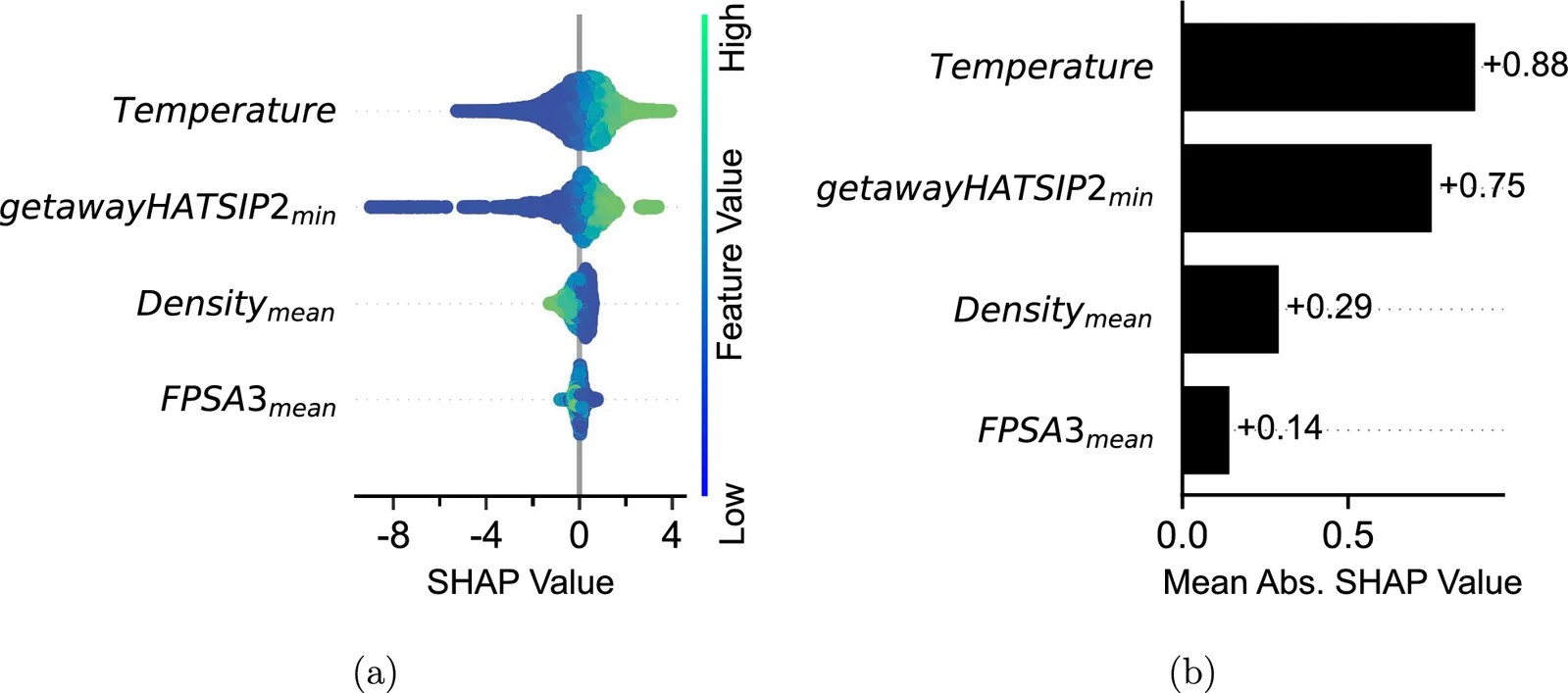
**a** Representative swarm of SHAP values and **b** mean absolute SHAP values. Each row corresponds to a feature of the model. In (**a**), color indicates the feature value of the instance, with green colors indicating higher feature values and blue colors indicating lower ones. Negative SHAP values correspond to a decrease in the vapor pressure prediction. In (**b**), the mean of absolute SHAP values indicate the overall influence of the feature. In both (**a**) and (**b**), the rows are descending in order of importance, where the most important features are listed first

To understand how each feature influences vapor pressure predictions, SHapley Additive exPlanations (SHAP) [70] values were computed and visualized in Fig. 5a. The mean absolute SHAP values and corresponding SHAP swarm plot quantify each feature’s contribution to the model output. According to the mean absolute SHAP values, the features contribute in the following order of importance: *Temperature* > ${getawayHATSIP2}_{min}$ > ${Density}_{mean}> {FPSA3}_{mean}$.

In the swarm plot in Fig. 5a, negative and positive SHAP values correspond lower and higher predicted vapor pressures, respectively. The colors represent the feature values in relative terms. Simple features such as *Temperature* and ${Density}_{mean}$ follow physical intuition: lower temperatures and higher densities correspond to lower vapor pressures. In contrast, the ${getawayHATSIP2}_{min}$ and ${FPSA3}_{mean}$ features are less straightforward to interpret. The ${getawayHATSIP2}_{min}$ feature is derived from HATS autocorrelation [71, 72] with a lag of two and weighted by ionization potential. The ${FPSA3}_{mean}$ feature is computed from a molecule’s solvent-accessible surface area (SASA) for atoms having positive partial charge divided by total SASA [73, 74]. The subsequent analysis of these features did not consider the influence of fluorine due to its small contribution to the dataset.

From Fig. 5a Lower ${FPSA3}_{mean}$ values correspond to higher vapor pressures, and high ${FPSA3}_{mean}$ values cluster near zero SHAP, indicating a neutral influence on predicting vapor pressure. However, intermediate ${FPSA3}_{mean}$ values correspond to lower vapor pressures. Chemically, high ${FPSA3}_{mean}$ values are typical of pure hydrocarbons, where numerous $\delta ^{+}$ hydrogens contribute to positively charged SASA. Intermediate ${FPSA3}_{mean}$ values arise from the replacement of only a few, as opposed to all, hydrogen atoms by $\delta ^{-}$ oxygen atoms in hydroxyl functional groups. The chemical understanding of the ${FPSA3}_{mean}$ feature connects molecular geometry and surface polarity to vapor pressure through intermolecular cohesion. The ${getawayHATSIP2}_{min}$ feature also considers electron behavior, but through an ionization potential weighting scheme instead of surface polarity.

In general, interpretation of HATS autocorrelation features are based on combining leverage distances and weighting schemes. For leverage distances, atoms located farther from the geometric center contribute increase the value of the feature than those close to the geometric center. Here, the geometric center is defined by 3D atomic coordinates irrespective of mass. The leverages are then weighted: for a weighting scheme utilizing atomic ionization potentials (O > H > C), oxygen atoms far from the center will increase the value significantly more than carbon or hydrogen. Additionally, because the HATS autocorrelation is summation based, more atoms are expected to further increase the feature value. This means that the ${getawayHATSIP2}_{min}$ feature is sensitive to both molecular size, shape, and weighting scheme. However, more interpretation approaches for HATS features still need to be developed [75]. Because interpretation of HATS descriptors remains a challenge, the GNN models provide a complementary perspective through substructure-level attribution analyses.

**Fig. 6:**
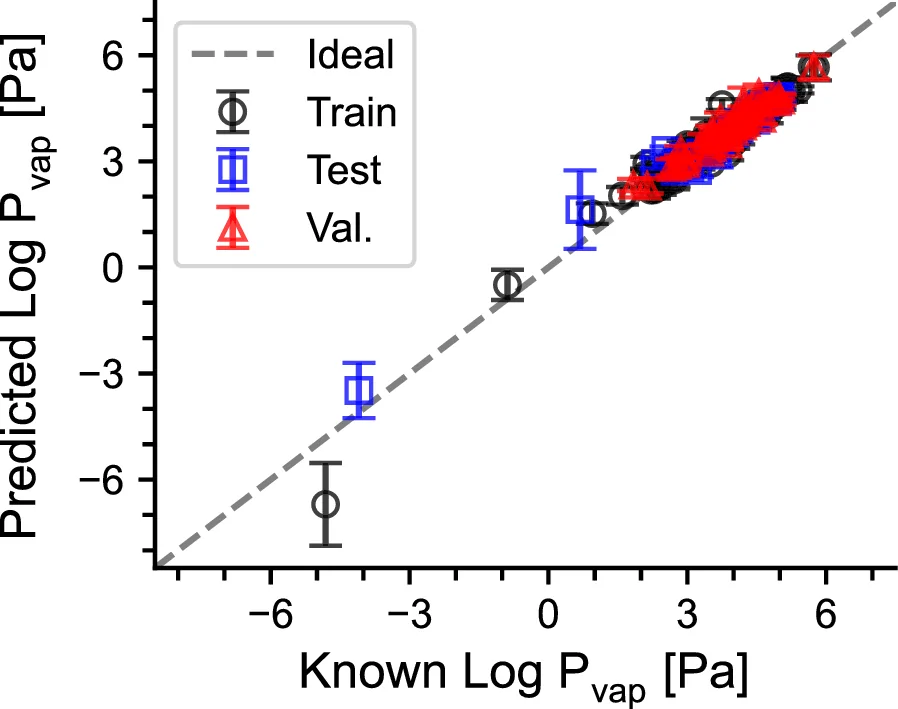
Parity plot of log vapor pressure ($P_{vap}$). The symbols and error bars represent the mean and standard deviation as predicted by the ensemble of ten RCGN models. Each marker is a unique molecule from the fixed-temperature dataset, where vapor pressure was calculated at 387 K

The parity plot of the GNN consensus model (of ten randomly seeded RCGN models) is shown in Fig. 6. The GNN consensus model is able to predict the bulk of the data; deviation plot in Figure S6. For the few low vapor pressure molecules in the dataset, there was a larger variance in the prediction, but the mean prediction remains close to the known value. The R^2^, RMSE, MAE, and MAPE were 0.95, 0.23 Pa, 0.16 Pa, and 4.8% for the training set, 0.91, 0.25 Pa, 0.20 Pa, and 5.7% for the validation set, and 0.96, 0.31 Pa, 0.22 Pa, and 9.7% for the test set, respectively.

**Fig. 7:**
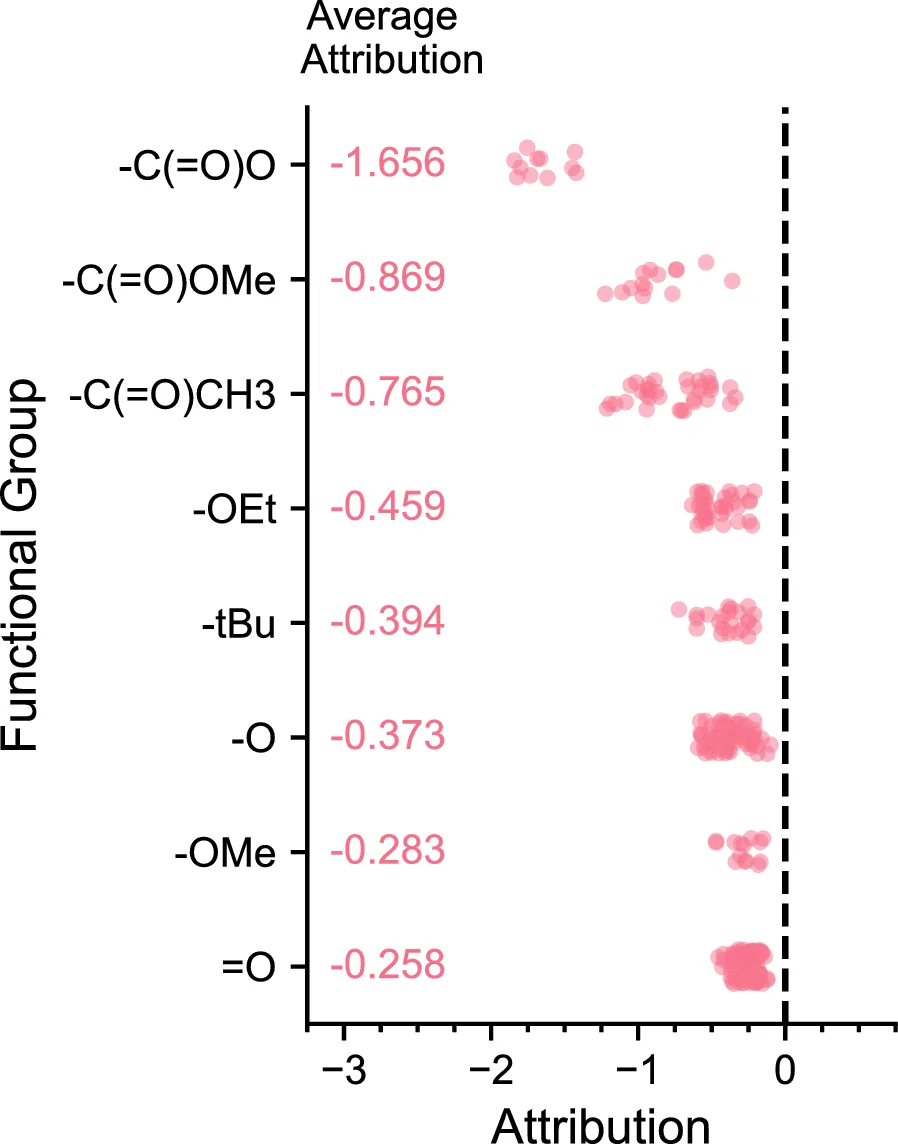
Swarm of functional group attribution to the predicted vapor pressure

The substructure masking reveals the key connections between molecular structure and vapor pressure. The attribution of functional groups (non-fluorinated) is shown in Fig. 7. Generally, the results show that the inclusion of functional groups contributed to lower vapor pressures. In particular, carboxylic acid, methyl ester, and methyl ketone functional groups were attributed with decreasing vapor pressure the most.

### Virtual screening

The GPR and GNN ensemble models were developed to guide the generation of new molecules and predict their vapor pressure. The interpretation of these models informed the inclusion of specific functional groups. For example, Fig. 7 indicated that functional groups such as carboxylic acid, methyl ester, and methyl ketone are associated with reduced vapor pressure, and these groups were therefore included in molecule generation. The molecule generation excluded fluorine to avoid the issues with additive compatibility and regulatory limitations of some fluorinated lubricants.

The generated molecules were created through a probabilistic growth procedure. Seed structures were selected with equal probability between linear alkyl chains and cyclic structures. Molecules were then “grown” by replacing a randomly chosen hydrogen with either a cyclic structure (5% probability), functional group (0 or 2% probability) or an alkyl chain. Growth stopped when the molecule either reached a molecular weight beyond 600 amu or 200 atoms. The number of carbon atoms in the additional alkyl chain was drawn from a Gaussian distribution (μ = 7, σ = 3), bounded between 3 and 12 carbon atoms. The cyclic structure could have been as a cyclohexane, cyclopentane, benzene, toluene, and naphthalene.

The generated molecules were then evaluated for synthetic accessibility using two established metrics: the Synthetic Accessibility Score (SAScore)  [76] and the SYnthetic Bayesian Accessibility (SYBA) score  [77]. The SAScore ranges from 1 (easily synthesizable) to 10 (difficult to synthesize). Although no strict threshold is prescribed in the original formulation, molecules with values above 6 are generally considered challenging to prepare  [76]. So, a cutoff of SAScore < 5 was applied to identify candidates within a readily synthesizable regime. The SYBA score provides a Bayesian classification of synthetic feasibility, where positive values are predicted to be easy-to-synthesize and the magnitude reflects confidence in that prediction  [77]. A threshold of SYBA > 0 was used to retain molecules predicted to be synthetically accessible.

The vapor pressure of the remaining generated molecules was predicted using both the GPR and GNN ensemble models and benchmarked against the MAC molecules. The GNN ensemble, trained to predict vapor pressure at 387 K, served as the initial filter. At 387 K, MAC lubricants have vapor pressures near $10^{-5}$ Pa, so generated molecules with GNN-predicted vapor pressures higher than $10^{-5}$ Pa were removed from the candidate pool.

The remaining molecules were then evaluated using the GPR ensemble, which can predict vapor pressure across a range of temperatures. However, MD simulations at multiple temperatures are computationally expensive and were only performed at 300 K, a recommended temperature for reporting vapor pressure experimentally [78]. At 300 K, MAC lubricants have a vapor pressure on the order of $5\times 10^{-9}$ Pa [6, 18, 55–58], so molecules with GPR-predicted vapor pressures above $5\times 10^{-9}$ Pa were removed from the candidate pool. From this process, more than 4500 molecules were identified with low vapor pressure for future studies.

To explore structural diversity among the generated molecular candidates, the molecules were projected onto a low-dimensional chemical space and grouped according to structural similarity. This process involved encoding molecular structures as Morgan fingerprints. Pairwise structural dissimilarities were calculated using the Rogers-Tanimoto distance calculated from Morgan fingerprints [79, 80]. The dissimilarities were then used as input for t-distributed Stochastic Neighbor Embedding (t-SNE) [81]. The two-dimensional t-SNE projection positioned structurally similar molecules close together, allowing visualization of chemical diversity and structure–property relationships.

**Fig. 8:**
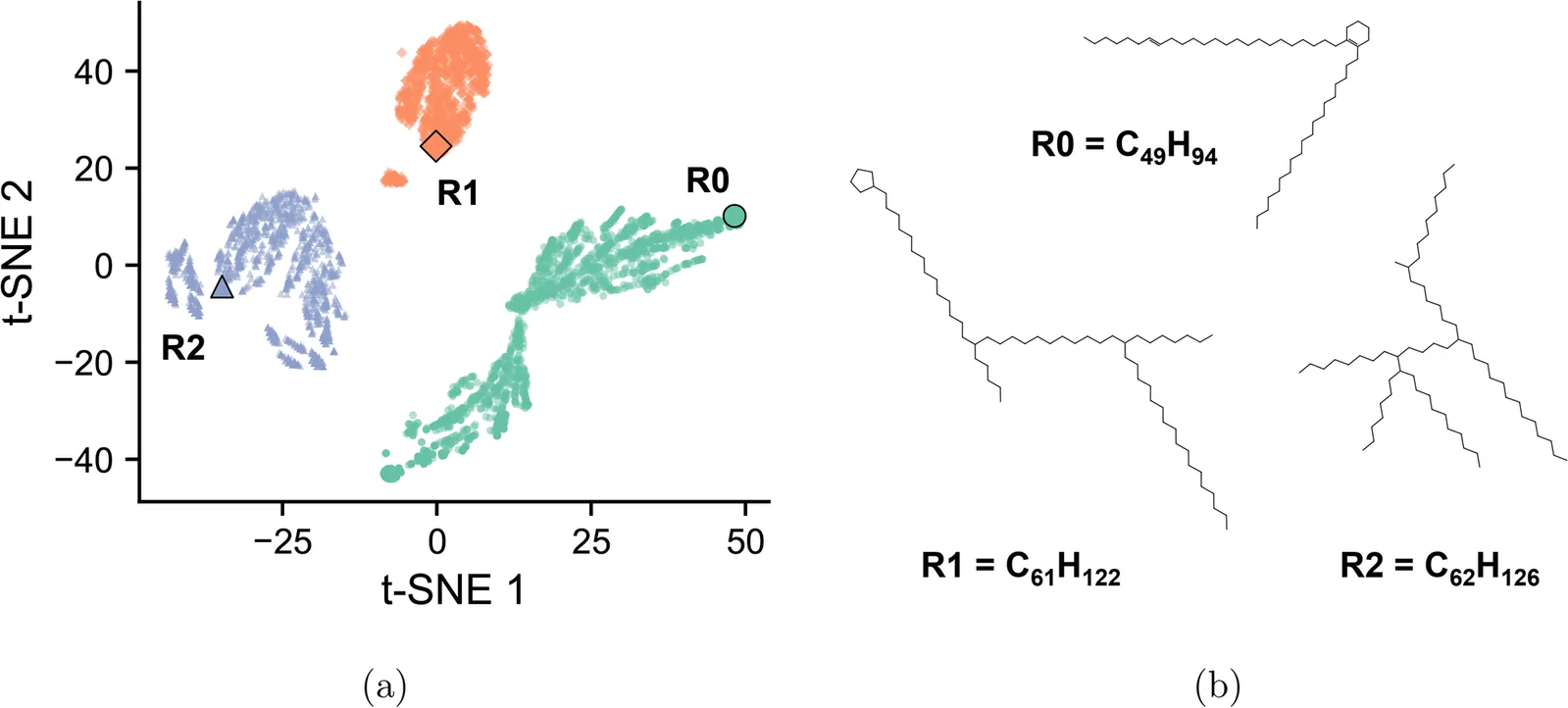
**a** t-SNE visualization of the chemical space and **b** representative low vapor pressure candidate molecular structures. In (**a**), the colors marker corresponds to clusters identified by DBSCAN algorithm. In (**b**), the representative molecular structures of each cluster are labeled with the molecular formula

Three distinct clusters within the projected space were identified using the Density Based Spatial Clustering of Applications with Noise (DBSCAN) algorithm [82, 83]. Figure 8a shows the t-SNE projection of low vapor pressure candidate molecular structures, clustered and distinguished by marker shape and color. Clusters 0, 1, and 2 contain 1821, 799, and 1345 molecules, respectively, which provides a large pool of candidate low vapor pressure molecular structures. The median vapor pressures of the three clusters are $1.3\times 10^{-9}$ Pa, $9.2\times 10^{-10}$ Pa, and $9.7\times 10^{-10}$ Pa for cluster 0, 1 and 2, respectively. The standard deviation of vapor pressure in all clusters was less than 0.25 in $\log _{10}$ space. From the clusters, a representative molecule for each cluster was determined by calculating pairwise Rogers-Tanimoto similarity among all cluster members. The molecule with the highest mean similarity to all others was identified as the central or representative structure for each cluster.

The three representative molecules, R0, R1, and R2, are shown along with their chemical formulas in Fig. 8b. R0 represents a set of molecules that largely contain linear hydrocarbons with some H/C functional groups, double bonds or a ring. The R1 molecule represents a set of longer chain molecules that may or may not contain cyclic structures but more branched than those in cluster 0. Finally, the R2 molecule represents a set of highly branched molecules that do not usually contain rings. Structurally, the low vapor pressure 1,3-bis(2-octyldodecyl) cyclopentane and 1,3,4-tri-(2-octyldodecyl) cyclopentane are similar to the R1 and R2 molecules. The R0 is similar to waxy molecules, such as paraffin waxes [84], which are known to have high melting points and may not be suitable as space lubricants in low temperature applications. Although these are representative molecules, each cluster contains a distribution of chemically similar structures and properties. This analysis suggests chemistries that are likely to have low vapor pressure and thus be candidates for subsequent investigation of their viability as space lubricants.

## Conclusions

This work demonstrated the integration of interpretable machine learning methods with molecular simulation data towards the discovery of space-compatible liquid-based lubricants. GPR and GNN ensemble models were developed that can predict vapor pressure across diverse chemical structures with high accuracy.

The GPR models captured quantitative relationships between temperature, molecular density, and electronic features, while the GNN models provided structural attributions that highlighted the effects of functional groups such as carboxylic acids and esters in reducing vapor pressure. The interpretability of these models provided valuable chemical insight into how molecular geometry, polarity, and composition influence volatility. Through virtual screening and clustering of generated molecules, a diverse set of candidates with predicted low vapor pressures was identified, which is the critical first step toward extending the range of candidate chemistries beyond existing PAO, PFPE and MAC lubricants.

In future research, candidate molecules should be evaluated in greater depth with respect to synthetic feasibility, for example through retrosynthetic analysis or reaction prediction methods, and with respect to additional space lubricant-relevant properties including radiation stability, thermal and oxidative stability, and viscosity-temperature behavior. More generally, the workflow developed here provides a generalizable and explainable approach for molecular property prediction and materials discovery in extreme environments.

## Supplementary Information

Supplementary file 1

## Data Availability

The data used in this study is available on the Github page: https://github.com/Dmiliate2/ML4VP.

## Code availability

Code used in this study is available on the Github page: https://github.com/Dmiliate2/ML4VP.

## References

[1] Fusaro RL (1994) Lubrication of space systems. NASA Lewis Research Center,(NASA/TM-106392 Technical Report)

[2] Krishnan S (2015) Lubrication for Moving Mechanical Systems Used in Spacecraft. Appl Mech Mater 772:446–452

[3] Jones Jr WR, Jansen MJ (2000) *Space Tribology*, NASA Glenn Research Center Technical Reports Technical Report NASA/TM-2000-209924

[4] Buttery M (2010) Proceedings of the 40th Aerospace Mechanisms Symposium

[5] Lu R, Mori S, Kubo T, Nanao H (2009) Effect of sulfur-containing additive on the decomposition of multialkylated cyclopentane oil on the nascent steel surface. Wear 267:1430–1435

[6] Venier CG, Casserly EW, Jones WR, Jr M, Marchetti MJ, Jansen RE, Predmore (2002) Proceedings of the 36th Aerospace Mechanisms Symposium

[7] Carré DJ (1988) The performance of perfluoropolyalkylether oils under boundary lubrication conditions. Tribol Trans 31:437–441

[8] Marchetti M, Sicre J, Jones WR, J (2002) Relative Lifetimes of MAPLUB Greases for Space Applications. NASA Glenn Research Center,(NAS 1.15:211875 Technical Report)

[9] Fusaro RL, Khonsari MM (1992) Liquid lubrication for space applications. NASA Lewis Research Center Technical Report

[10] Scheidler J, Stalcup E, Montbach E (2022) The impacts of heating actuators in extremely cold space environments. IEEE Aerosp Conf (AERO) 2022:1–8

[11] Kent A, Bingley R, Renondeau H, Vortselas A, Romera F (2023) 20th European Space Mechanisms and Tribology Symposium, p. 8

[12] Jones WR Jr, Jansen MJ (2008) Proceedings of the institution of mechanical engineers. Part J J Eng Tribol 222:997–1004

[13] Jones Jr WR, Jansen MJ (2006) *Handbook of lubrication and tribology: Volume I application and maintenance, second edition*, CRC Press, Boca Raton, pp. 27–1–27–41

[14] Campbell WA, Jr RS, Marriott JJ, Park (1984) Outgassing Data for Selecting Spacecraft Materials, NASA Goddard Space Flight Center.(NAS 1.61:1124 Technical Report)

[15] ASTM International, *ASTM E595 - 15 - Standard Test Method for Total Mass Loss and Collected Volatile Condensable Materials from Outgassing in a Vacuum Environment*, (2021)

[16] (2008) European Cooperation for Space Standardization, ECSS-Q-ST-70-02C—Thermal vacuum outgassing test for the screening of space materials

[17] Jacobson NS, Kowalski BA (2020) Thermodynamic Measurements Using the Knudsen Cell Technique, National Aeronautics and Space Administration Glenn Research Center.(NASA/TM-2020-220495 Technical Report)

[18] Nguyen QN, Jones WR Jr (2001) Volatility and wear characteristics of a variety of liquid lubricants for space applications. Tribol Trans 44:671–677

[19] Knudsen M (1909) Effusion and the molecular flow of gases through openings. Ann Phys 333:999–1016

[20] Oja V, Suuberg EM (1997) Development of a nonisothermal Knudsen effusion method and application to PAH and cellulose tar vapor pressure measurement. Anal Chem 69:4619–4626

[21] Magomedov MN (2023) On the accuracy of the Clausius–Clapeyron relation. Vacuum 217:112494

[22] Brown OLI (1951) The Clausius–Clapeyron equation. J Chem Educ 28:428

[23] Thompson JE, Paluch AS (2023) Revisiting the Clausius/Clapeyron equation and the cause of linearity. Thermo 3:412–423

[24] Moshele P, Stenzel MR, Drolet D, Arnold SF (2024) Comparing Antoine parameter sources for accurate vapor pressure prediction across a range of temperatures. Ann Work Expo Health 68:409–419 10.1093/annweh/wxae010 PMC11033562 38437526

[25] Wisniak J, Ph J (2001) Historical development of the vapor pressure equation from Dalton to Antoine. Equilibria 22:622–630

[26] Factorovich MH, Molinero V, Scherlis DA (2014) A simple grand canonical approach to compute the vapor pressure of bulk and finite size systems. J Chem Phys 140:064111 10.1063/1.4865137 24527904

[27] Rane KS, Murali S, Errington JR (2013) Monte Carlo simulation methods for computing liquid-vapor saturation properties of model systems. J Chem Theory Comput 9:2552–2566 10.1021/ct400074p 26583852

[28] Hens R, Vlugt TJH (2018) Molecular simulation of vapor-liquid equilibria using the Wolf method for electrostatic interactions. J Chem Eng Data 63:1096–1102 10.1021/acs.jced.7b00839 PMC6150682 30258248

[29] Eggimann BL, Sun Y, DeJaco RF, Singh R, Ahsan M, Josephson TR, Siepmann JI (2020) Assessing the quality of molecular simulations for vapor–liquid equilibria: an analysis of the TraPPE database. J Chem Eng Data 65:1330–1344

[30] Muniz MC, Gartner TE 3rd, Riera M, Knight C, Yue S, Paesani F, Panagiotopoulos AZ (2021) Vapor-liquid equilibrium of water with the MB-pol many-body potential. J Chem Phys 154:211103 10.1063/5.0050068 34240989

[31] Yakubovich AV, Son W-J, Kwon O, Choi H, Choi B, Kim S (2020) Accurate vapor pressure prediction for large organic molecules: application to materials utilized in organic light-emitting diodes. J Chem Theory Comput 16:5845–5851 10.1021/acs.jctc.9b01245 32786920

[32] Quoika PK, Zacharias M (2024) Liquid-vapor coexistence and spontaneous evaporation at atmospheric pressure of common rigid Three-Point water models in molecular simulations. J Phys Chem B 128:2457–2468 10.1021/acs.jpcb.3c08183 PMC10945489 38427971

[33] Clausius R (1870) On a mechanical theorem applicable to heat. Lond Edinb Dublin Philos Mag J Sci 40:122–127

[34] Tsai DH (1979) The virial theorem and stress calculation in molecular dynamics. J Chem Phys 70:1375–1382

[35] Basak SC, Gute BD, Grunwald GD (1997) Use of topostructural, topochemical, and geometric parameters in the prediction of vapor pressure: a hierarchical QSAR approach. J Chem Inf Comput Sci 37:651–655

[36] Liang C, Gallagher DA (1998) QSPR prediction of vapor pressure from solely theoretically-derived descriptors. J Chem Inf Comput Sci 38:321–324

[37] McClelland HE, Jurs PC (2000) Quantitative structure-property relationships for the prediction of vapor pressures of organic compounds from molecular structures. J Chem Inf Comput Sci 40:967–975 10.1021/ci990137c 10955525

[38] Katritzky AR, Wang Y, Sild S, Tamm T, Karelson M (1998) QSPR studies on vapor pressure, aqueous solubility, and the prediction of Water–Air partition coefficients. J Chem Inf Comput Sci 38:720–725

[39] Goll ES, Jurs PC (1999) Prediction of vapor pressures of hydrocarbons and halohydrocarbons from molecular structure with a computational neural network model. J Chem Inf Comput Sci 39:1081–1089

[40] Basak SC, Mills D (2001) Quantitative structure-property relationships (QSPRs) for the estimation of vapor pressure: a hierarchical approach using mathematical structural descriptors. J Chem Inf Comput Sci 41:692–701 10.1021/ci000165r 11410048

[41] Panwar P, Yang Q, Martini A (2024) Temperature-dependent density and viscosity prediction for hydrocarbons: machine learning and molecular dynamics simulations. J Chem Inf Model 64:2760–2774 10.1021/acs.jcim.3c00231 37582234

[42] Santana VV, Rebello CM, Queiroz LP, Ribeiro AM, Shardt N, Nogueira IBR (2024) PUFFIN: a path-unifying feed-forward interfaced network for vapor pressure prediction. Chem Eng Sci 286:119623

[43] Lin Y-H, Liang H-H, Lin S-T, Li Y-P (2024) *J. Taiwan Inst. Chem. Eng.*, 105926

[44] Hoffmann M, Hasse H, Jirasek F (2025) GRAPPA–A hybrid graph neural network for predicting pure component vapor pressures Chem Eng J Adv. 100750

[45] Qu C, Kearsley AJ, Schneider BI, Keyrouz W, Allison TC (2022) Graph convolutional neural network applied to the prediction of normal boiling point. J Mol Graph Model 112:108149 10.1016/j.jmgm.2022.108149 35149486

[46] Wang G, Hu P (2023) Prediction of normal boiling point and critical temperature of refrigerants by graph neural network and transfer learning. Int J Refrig 151:97–104

[47] Wu Z, Wang J, Du H, Jiang D, Kang Y, Li D, Pan P, Deng Y, Cao D, Hsieh C-Y, Hou T (2023) Chemistry-intuitive explanation of graph neural networks for molecular property prediction with substructure masking. Nat Commun 14:2585 10.1038/s41467-023-38192-3 PMC10160109 37142585

[48] Wong WA, Wilson S, Collins J, Wilson K (2015) 13th International Energy Conversion Engineering Conference. Reston, Virginia

[49] Linstrom P (1997) NIST Standard Reference Database 69:IST Chemistry WebBook

[50] Becke AD (1993) A new mixing of Hartree–Fock and local density-functional theories. J Chem Phys 98:1372–1377

[51] Lee C, Yang W, Parr RG (1988) Development of the Colle–Salvetti correlation-energy formula into a functional of the electron density. Phys Rev B: Condens Matter 37:785–789 10.1103/physrevb.37.785 9944570

[52] Becke AD (1988) Correlation energy of an inhomogeneous electron gas: a coordinate-space model. J Chem Phys 88:1053–1062

[53] Hehre WJ, Ditchfield R, Pople JA (1972) Self–consistent molecular orbital methods. XII. Further extensions of Gaussian–type basis sets for use in molecular orbital studies of organic molecules. J Chem Phys 56:2257–2261

[54] Frisch MJ, Trucks GW, Schlegel HB, Scuseria GE, Robb MA, Cheeseman JR, Scalmani G, Barone V, Petersson GA, Nakatsuji H, Li X, Caricato M, Marenich AV, Bloino J, Janesko BG, Gomperts R,Mennucci B, Hratchian HP, Ortiz JV, Izmaylov AF, Sonnenberg JL, Williams-Young D, Ding F, Lipparini F, Egidi F, Goings J, Peng B, Petrone A, Henderson T, Ranasinghe D, Zakrzewski VG, Gao J,Rega N,Zheng G, Liang W, Hada M, Ehara M, Toyota K, Fukuda R, Hasegawa J, Ishida , Nakajima T, Honda Y, Kitao O, Nakai H, Vreven T, Throssell K, Montgomery Jr, Peralta JE, Ogliaro F, Bearpark MJ, Heyd JJ, Brothers EN,Kudin KN, Staroverov VN, Keith TA, Kobayashi R, Normand J, Raghavachari K, Rendell AP, Burant JC, Iyengar SS, Tomasi J, Cossi M, Millam JM, Klene M, Adamo , Cammi R, Ochterski JW, Martin RL, Morokuma K, Farkas O, Foresman JB,Fox DJ (2016) *Gaussian 16 Revision B.01*,

[55] Nye Lubricants Inc., *NYE SYNTHETIC OIL 2001*

[56] Jones WR Jr, Jansen MJ, Gschwender LJ, Snyder CE Jr, Sharma SK, Predmore RE, Dube MJ (2004) The tribological properties of several silahydrocarbons for use in space mechanisms. J Synth Lubr 20:303–315

[57] Venier CG, Jones Jr WR, Jansen MJ, Marchetti M (2003) *Proceedings of the 10th European Space Mechanisms and Tribology Symposium, 24-26 September 2003, San Sebastián, Spain. Compiled by R. A. Harris. ESA SP-524, Noordwijk, Netherlands: ESA Publications Division*, **524**, 337–340

[58] Nye Lubricants Inc (**NYE SYNTHETIC OIL 1001**)

[59] Weininger D (1988) SMILES, a chemical language and information system. 1. Introduction to methodology and encoding rules. J Chem Inf Comput Sci 28:31–36

[60] Landrum G, Tosco P, Kelley B, Rodriguez R, Cosgrove D, Vianello R, sriniker, Gedeck P,Jones G, NadineSchneider, Kawashima E, Nealschneider D,Dalke A, Swain M, Cole B, Turk S, Savelev A, Vaucher A, Wójcikowski M, Take I, tadhurst-cdd, Scalfani VF, Walker R,Ujihara K, Probst D, Lehtivarjo J, Godin G, Pahl A, Bérenger F,Faara H (2024) *rdkit/rdkit: 2024_09_4 (Q3 2024) Release*

[61] Panwar P, Yang Q, Martini A (2023) PyL3dMD: python LAMMPS 3D molecular descriptors package. J Cheminform 15:69 10.1186/s13321-023-00737-5 PMC10385924 37507792

[62] Jiang L, Greenwood CMT, Yao W, Li L (2020) Bayesian Hyper-LASSO classification for feature selection with application to endometrial cancer RNA-seq data. Sci Rep 10:9747 10.1038/s41598-020-66466-z PMC7297975 32546735

[63] Tibshirani R, Stat JR (1996) Regression shrinkage and selection via the lasso. Soc Series B Stat Methodol 58:267–288

[64] Inc. , The Mathworks, *MATLAB Version: 24.2.0.2712019 (R2024b)*, 2024

[65] Degen J, Wegscheid-Gerlach C, Zaliani A, Rarey M (2008) On the art of compiling and using ‘drug-like’ chemical fragment spaces. ChemMedChem 3:1503–1507 10.1002/cmdc.200800178 18792903

[66] Hu Y, Stumpfe D, Bajorath J (2016) Computational exploration of molecular scaffolds in medicinal chemistry. J Med Chem 59:4062–4076 10.1021/acs.jmedchem.5b01746 26840095

[67] Bemis GW, Murcko MA (1996) The properties of known drugs. 1. Molecular frameworks. J Med Chem 39:2887–2893 10.1021/jm9602928 8709122

[68] Lin Y-H, Liang H-H, Lin S-T, Li Y-P (2024) Advancing vapor pressure prediction: A machine learning approach with directed message passing neural networks. J Taiwan Inst Chem Eng 105926

[69] Hoffmann M, Hasse H, Jirasek F (2025) GRAPPA–a hybrid graph neural network for predicting pure component vapor pressures. Chem Eng J Adv 22:100750

[70] Lundberg SM, Lee S-I (2017) Neural Information Processing Systems. pp 4766–4777

[71] Consonni V, Todeschini R, Pavan M (2002) Structure/response correlations and similarity/diversity analysis by GETAWAY descriptors. 1. Theory of the novel 3D molecular descriptors. J Chem Inf Comput Sci 42:682–692 10.1021/ci015504a 12086530

[72] Consonni V, Todeschini R, Pavan M, Gramatica P (2002) Structure/response correlations and similarity/diversity analysis by GETAWAY descriptors. 2. Application of the novel 3D molecular descriptors to QSAR/QSPR studies. J Chem Inf Comput Sci 42:693–705 10.1021/ci0155053 12086531

[73] Stanton DT, Jurs PC (1990) Development and use of charged partial surface area structural descriptors in computer assisted quantitative structure property relationship studies. Anal Chem 62:2323–2329 10.1021/ci034284t 15154770

[74] Stanton DT, Egolf LM, Jurs PC, Hicks MG (1992) Computer-assisted prediction of normal boiling points of pyrans and pyrroles. J Chem Inf Comput Sci 32:306–316

[75] Zapadka M, Dekowski P, Kupcewicz B (2022) HATS5m as an example of GETAWAY molecular descriptor in assessing the similarity/diversity of the structural features of 4-thiazolidinone. Int J Mol Sci 23:6576 10.3390/ijms23126576 PMC9223869 35743020

[76] Ertl P, Schuffenhauer A (2009) Estimation of synthetic accessibility score of drug-like molecules based on molecular complexity and fragment contributions. J Cheminform 1:8 10.1186/1758-2946-1-8 PMC3225829 20298526

[77] Voršilák M, Kolář M, Čmelo I, Svozil D (2020) SYBA: Bayesian estimation of synthetic accessibility of organic compounds. J Cheminform 12:35 10.1186/s13321-020-00439-2 PMC7238540 33431015

[78] ASTM International (2025) ASTM E1194-25 Standard test method for vapor pressure

[79] Bajusz D, Rácz A, Héberger K (2015) Why is Tanimoto index an appropriate choice for fingerprint-based similarity calculations? J Cheminform 7:20 10.1186/s13321-015-0069-3 PMC4456712 26052348

[80] Rogers DJ, Tanimoto TT (1960) A computer program for classifying plants. Science 132:1115–1118 10.1126/science.132.3434.1115 17790723

[81] Orlov AA, Akhmetshin TN, Horvath D, Marcou G, Varnek A (2025) From high dimensions to human insight: exploring dimensionality reduction for chemical space visualization. Mol Inform 44:e202400265 10.1002/minf.202400265 PMC11733715 39633514

[82] Ester M, Kriegel H, Sander J, Xu X (1996) A density-based algorithm for discovering clusters in large spatial databases with noise. KDD 226–231

[83] Schubert E, Sander J, Ester M, Kriegel HP, Xu X (2017) DBSCAN revisited, revisited: why and how you should (still) use DBSCAN. Trans ACM Database Syst 42:1–21

[84] Kumar V, Pallapa M, Rezai P, Selvaganapathy PR (2016) Reference module in materials science and materials engineering. Elsevier

