# Supplementary material for: Computational design of low-volatility lubricants for space using interpretable machine learning
Source: J Cheminform. 2026 Jul 2;18:133. doi: 10.1186/s13321-026-01250-1 (PMC13602501; doi:10.1186/s13321-026-01250-1)
Supplement: Supplementary file 1 [file 13321_2026_1250_MOESM1_ESM.pdf]

Supplementary Information

Computational Design of Low-Volatility Lubricants for Space Using Interpretable Machine Learning

Daniel Miliate[1] and Ashlie Martini[1*]

[1]**Department of Mechanical & Aerospace Engineering, University of California Merced, Merced, CA 95343, United States**

A. MAC Data

Data is limited for very low vapor pressure substances, but it is available for 1,3,4-tri-(2-octyldodecyl) cyclopentane[S1–S5] and bis(2-octyldodecyl) cyclopentane[S3–S6] MAC lubricants. Data was extracted using WebPlotDigitizer[S7] when it was not tabulated. The colored markers in Figure S1 correspond to reported data, where the marker shape corresponds to the source. Parameters for Antoine's equation were then calculated from the reported data, excluding outliers for 1,3-bis(2-octyldodecyl)[S3]. From the fit lines, vapor pressures and corresponding temperatures were sampled for training in GPR and GNN models.

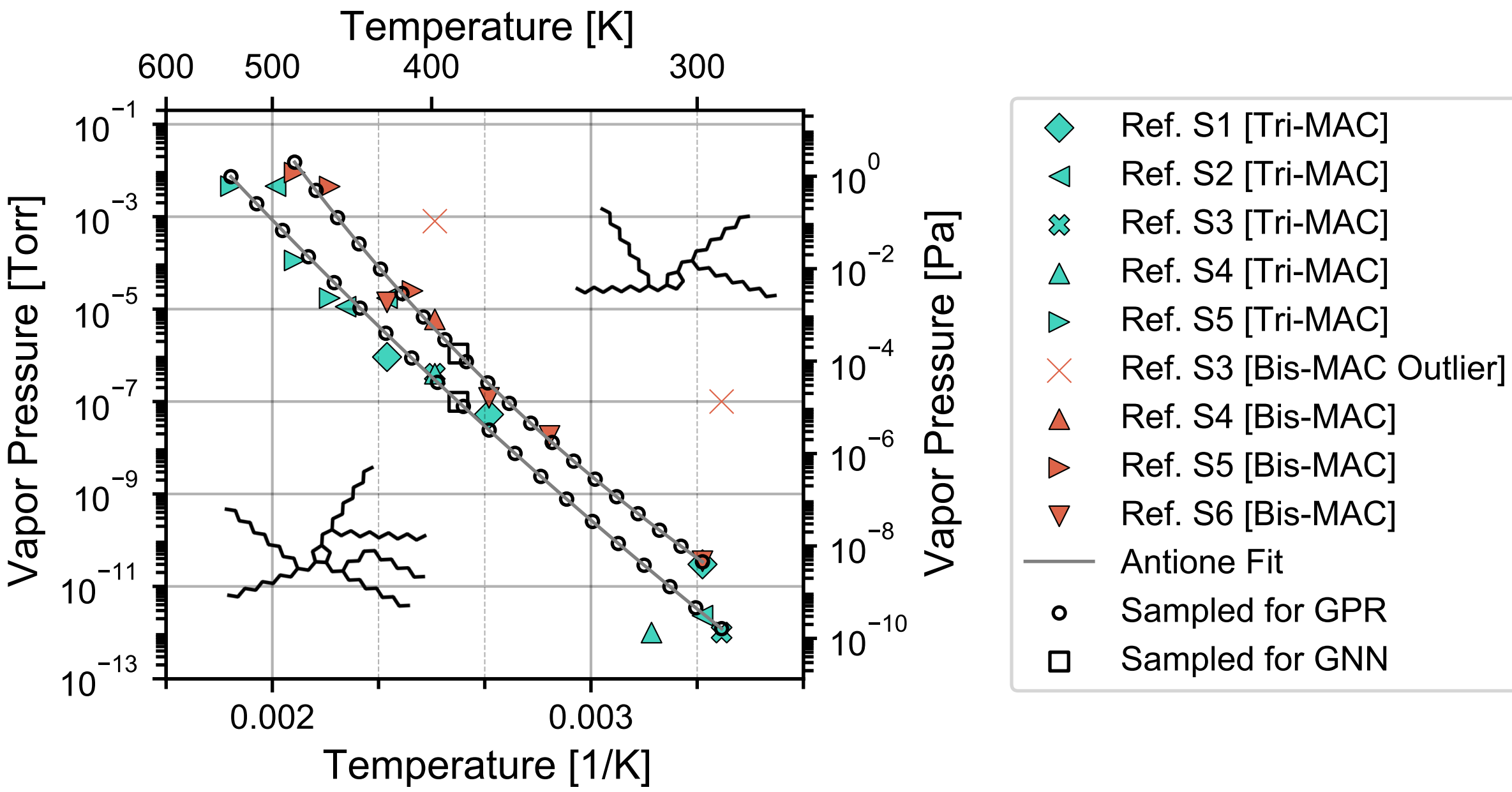

Figure S1: Vapor pressure vs temperature for 1,3,4-tri-(2-octyldodecyl) cyclopentane[S1–S5] and 1,3-bis(2-octyldodecyl) cyclopentane[S3–S6].

B. Vapor Pressure Temperature Range

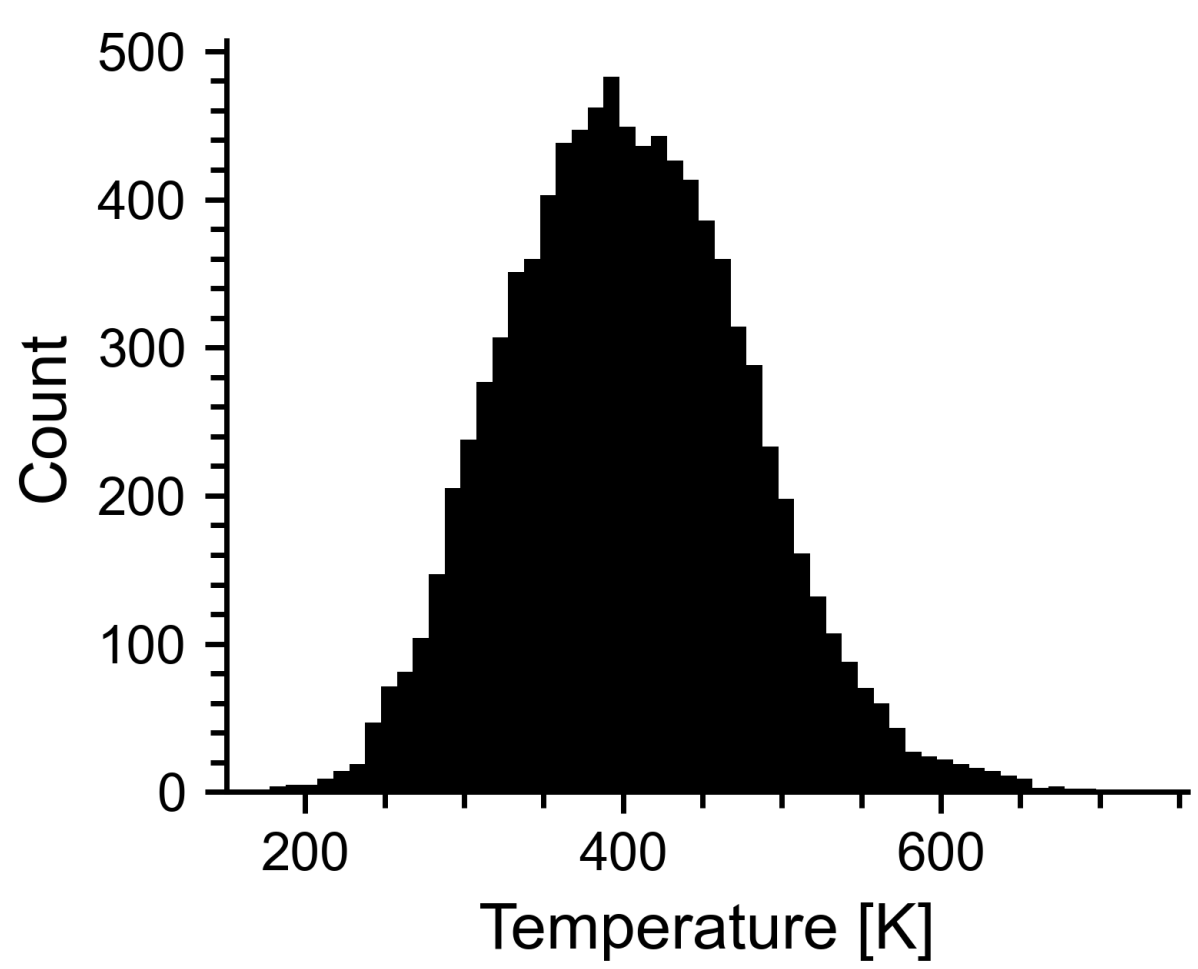

Figure S2: Distribution of the temperatures of vapor pressure data used in the GPR model development.

C. Molecular Dynamics Simulation Protocol

To generate high-throughput MD simulations, BOSS[S8] was used to convert SMILES strings to structure files with OPLS-AA forcefields[S9]. OPLS-AA forcefields are widely accepted for non-reactive MD simulations. The structure files were then used to build the simulation box with a maximum of 5000 atoms and 0.3 g/cc initial packing density, constructed by packmol[S10]. The simulations were performed using the Large Atomic/Molecular Massively Parallel Simulator (LAMMPS) opensource software package[S11]. The MD simulation protocol was designed for relatively low computational costs, using both canonical (NVT) and isothermal–isobaric (NPT) ensembles and timestep of 1 fs.

The MD simulation began with energy minimization, setting the stopping tolerance for energy (unitless) at 1.0e-16, stopping tolerance for force at 1.06e-6 (kcal/mol)/Å, a maximum of 100,000 iterations, and a maximum of 500,000 evaluations. The simulation box was held at constant volume and constant temperature 1000 K with NVT for 50 ps. Next, the box was held at 1 atm constant pressure and constant temperature with NPT for 150 ps, ensuring density reached steady state. The simulation temperature of this step was chosen to match the temperature used in calculating vapor pressure. Finally, at the same pressure and temperature the NPT simulation trajectory was sampled every 0.5 ps for 50 ps to calculate dynamic features. Figure S3b demonstrates the variation in MD descriptors used in training.

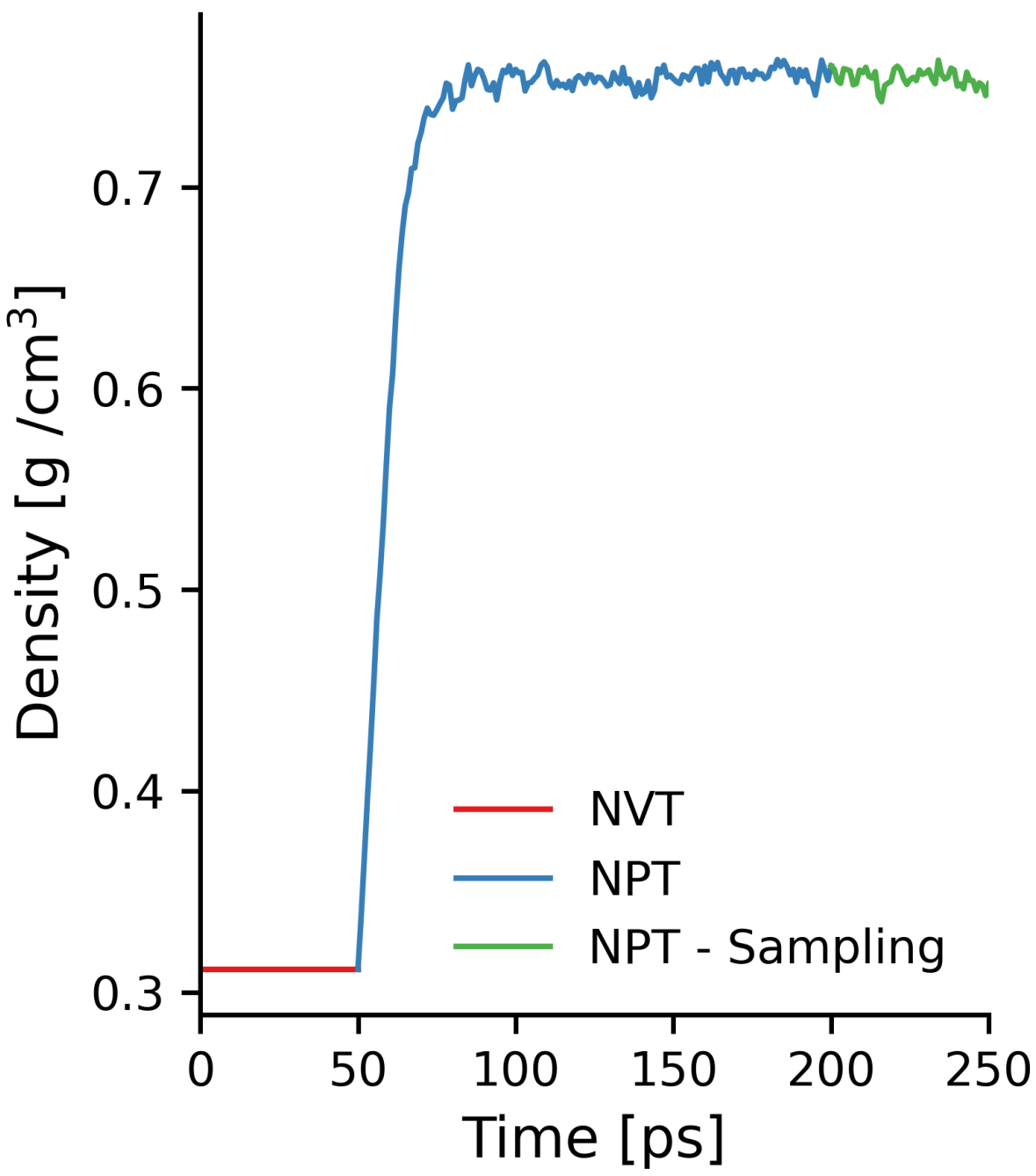

(a)

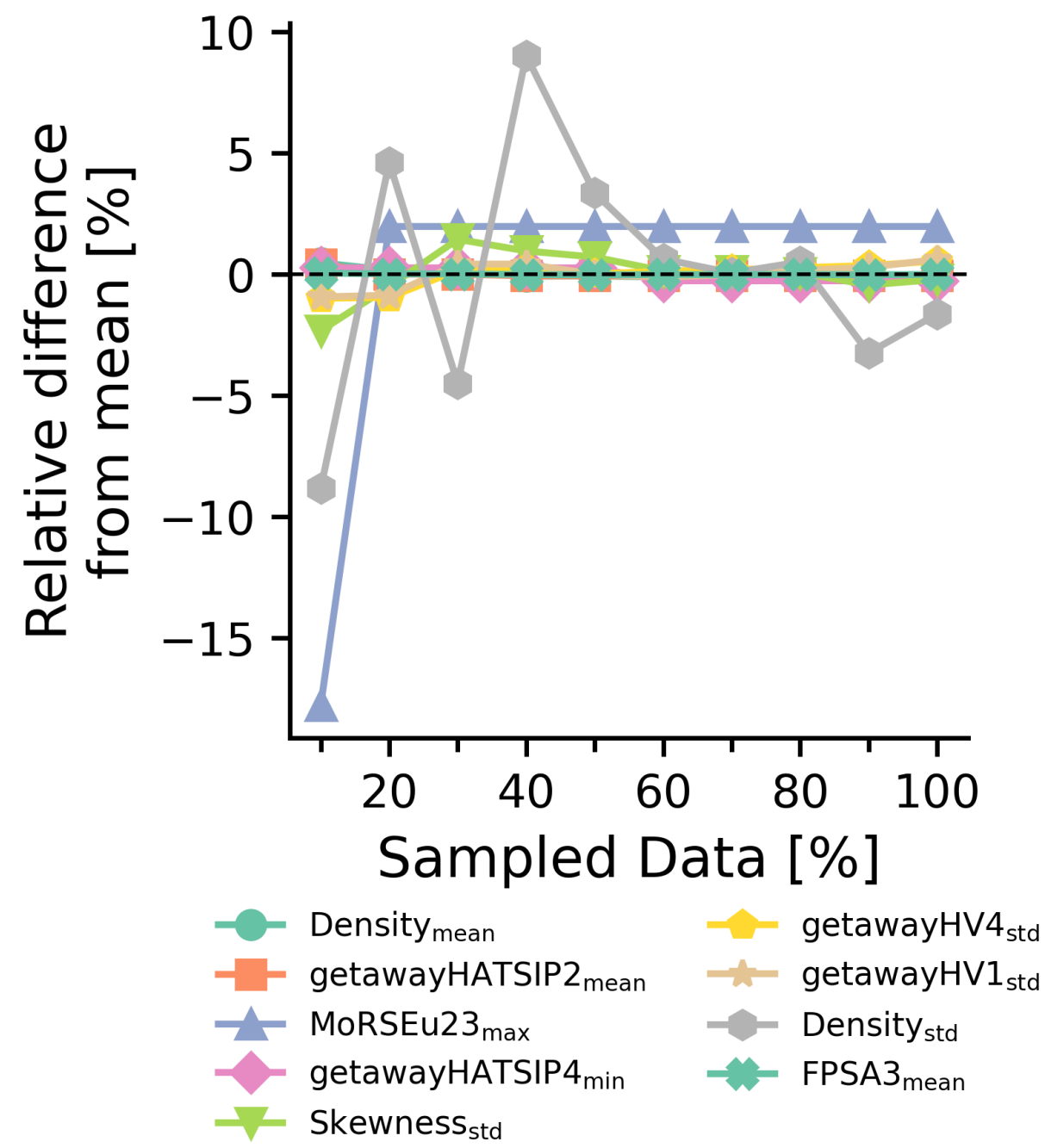

(b)

Figure S3: Representative (a) Density vs Time and (b) Relative change of MD derived features vs percent of sampling data. In (a), the density reached steady state well before the sampling step. In (b), the time dependent MD descriptors were calculated using increasing amounts of the 50 ps of sampled data. The top ten GPR models contained nine time dependent MD descriptors, which are shown. The relative difference between the MD descriptor with varying percent of sampled data was compared to the mean. In all cases, only about 25 ps of sampled data was required to reach less than 5 % difference.

D. Data Splitting

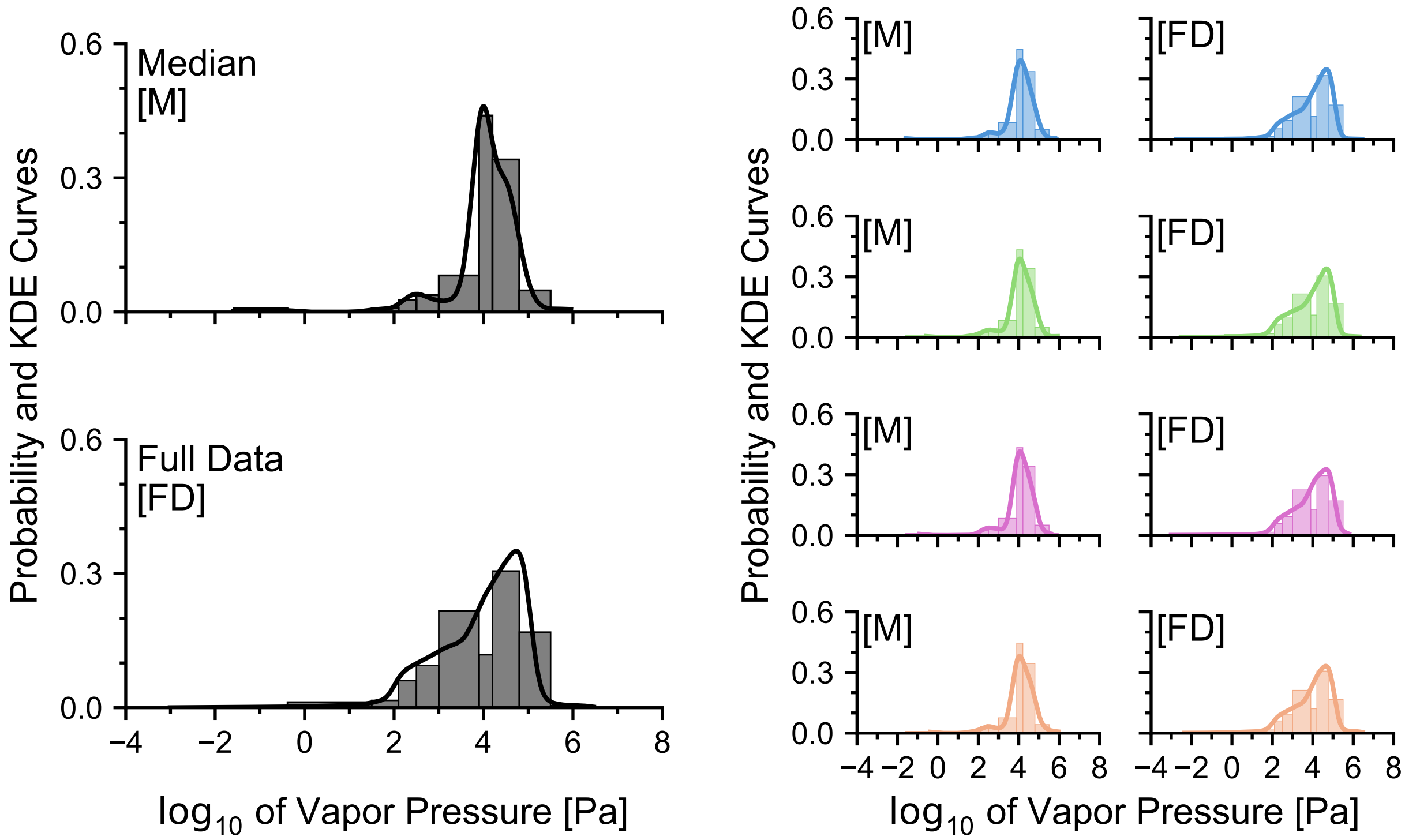

(a) (b)

Figure S4: Overview probability histograms and kernel density estimate curves of the dataset and partitions for the median-of-molecular-structure [M] and full dataset [FD] of vapor pressure. In (a), the top and bottom histograms show the probability for the median and full dataset, respectively. In (b), each row represents the split partitions to be used for train & validation sets of the outer CV and test set for final evaluation. The first and second columns represent the median-of-molecular-structure and full dataset, respectively.

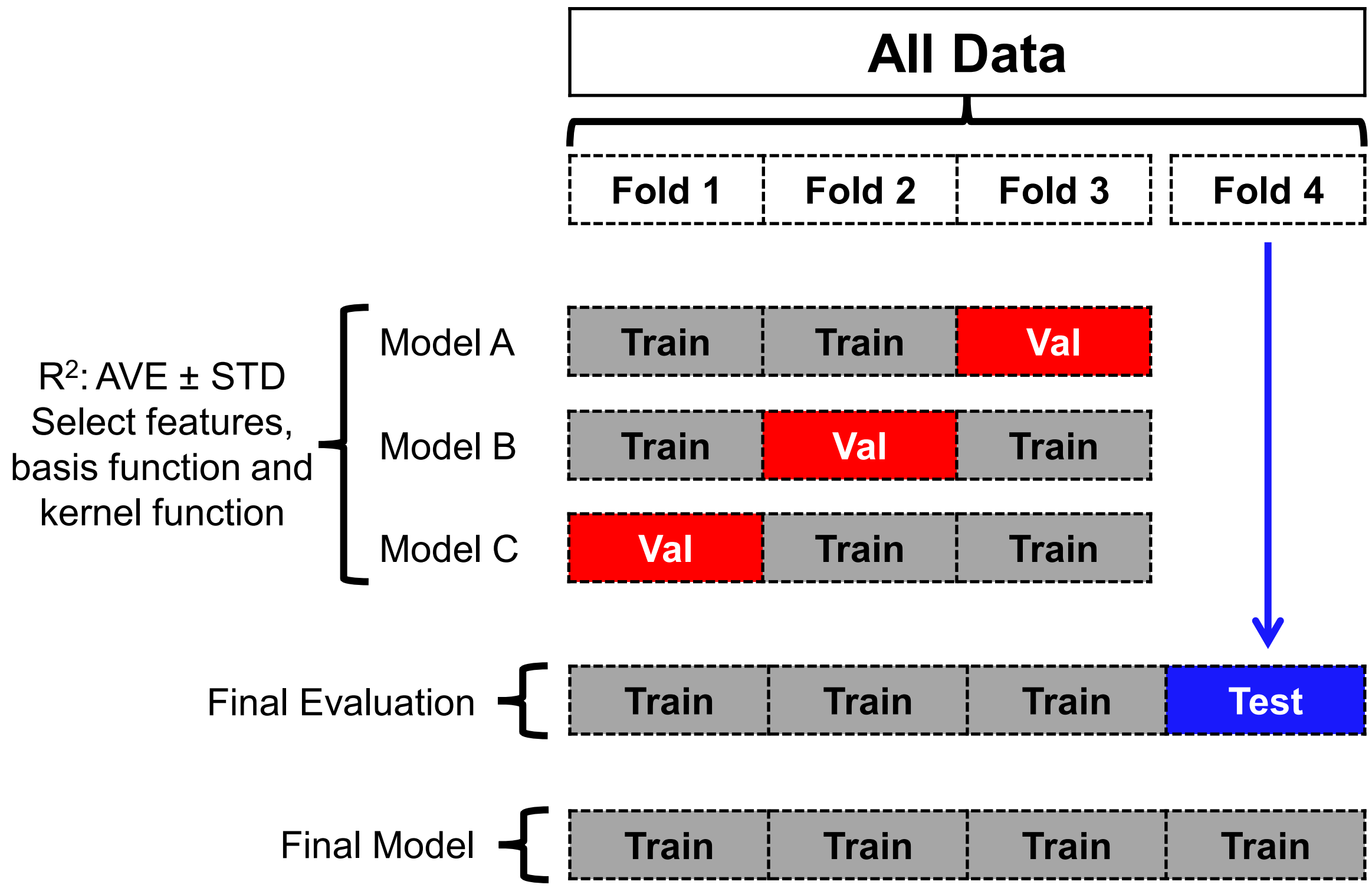

Figure S5: Data splitting workflow for nested CV model, final evaluation, and final model. During cross-validation, the best feature subsets, then basis and kernel functions are determined before final evaluation.

E. Model Performance

The GPR and GNN parity plots are recreated as deviation plots in Figures S6a and S6b, respectively. Additionally, the GPR model was compared to GRAPPA[S12] and Chemprop_VP[S13] using the holdout test set. GRAPPA was trained on over 20,000 compounds above 1 Pa. Using GRAPPA, vapor pressure was either predicted directly or through Antione's parameters. The GPR model performs better below 1kPa and the GRAPPA models perform better above 1 kPa. The Chemprop_VP model performs well but under predicts the lowest vapor pressure.

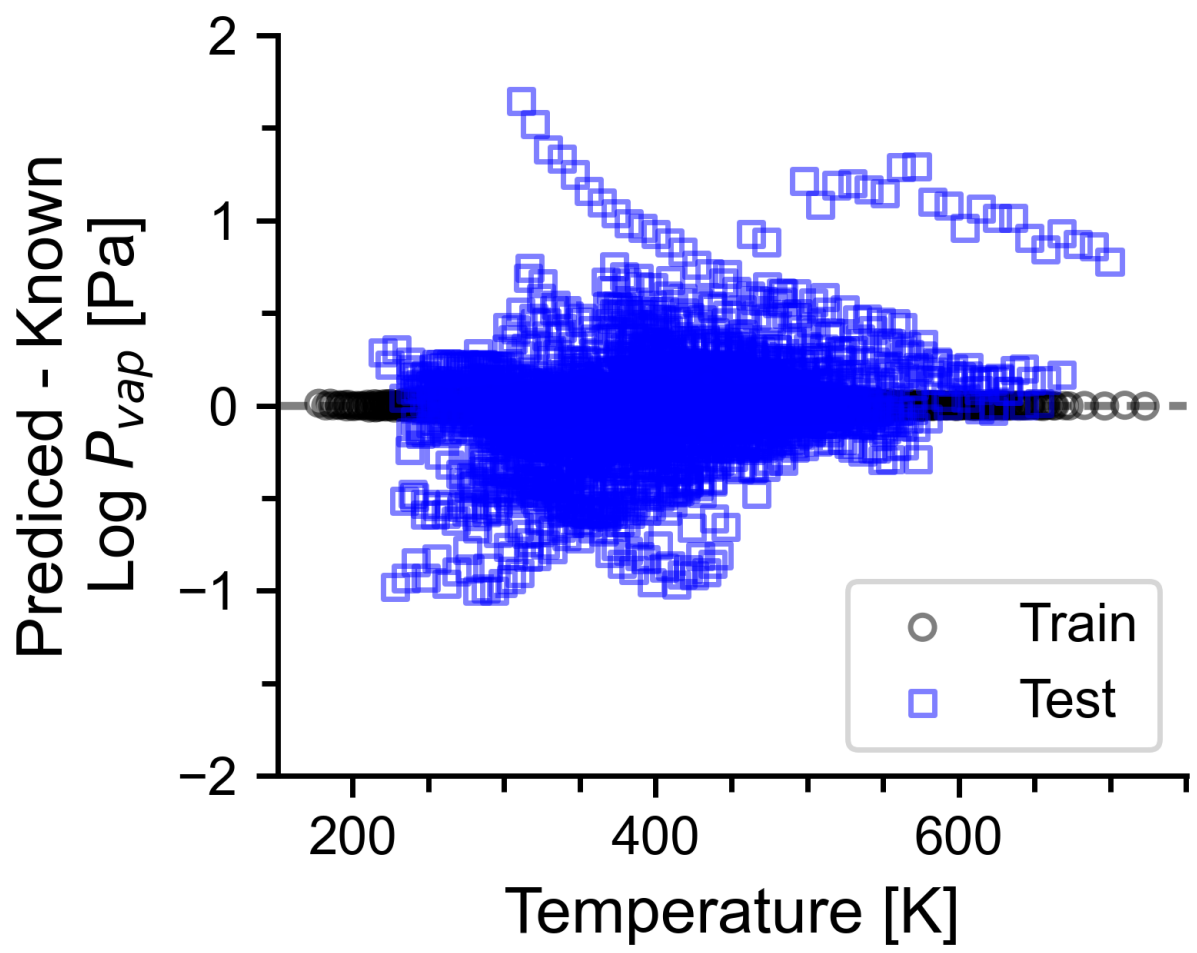

(a)

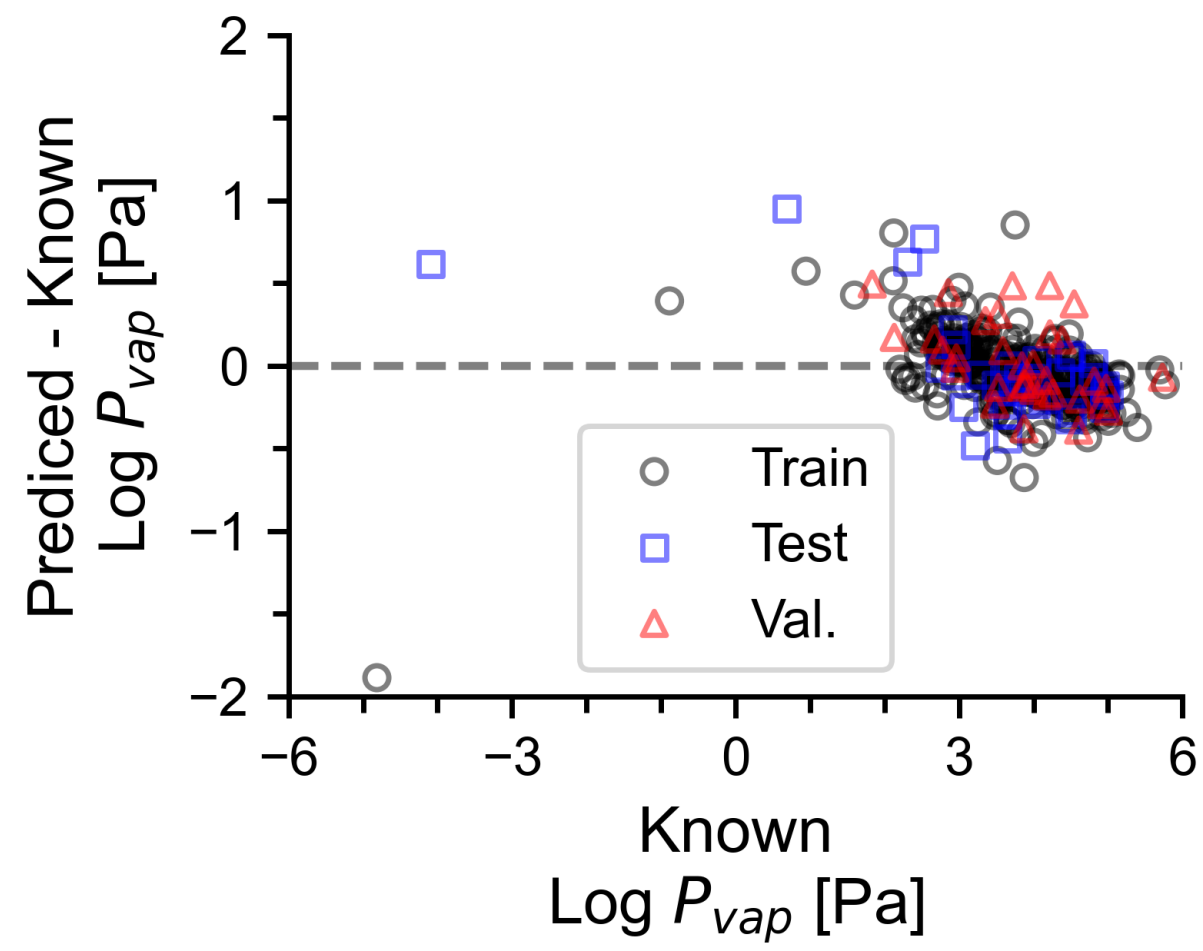

(b)

Figure S6: Deviation plots for the (a) GPR and (b) GNN models. In (a), the prediction error, predicted minus known, is plotted as a function of temperature. Because all GNN predictions are made at 387 K, temperature is not a variable in (b); instead, the prediction error is plotted against the known vapor pressure.

Model #	Training Split				Test Split			
	R^2	RMSE (Pa)	MAE (Pa)	MAPE (%)	R^2	RMSE (Pa)	MAE (Pa)	MAPE (%)
1	0.9999	0.0007	0.0004	0.0139	0.9403	0.3116	0.2306	7.4090
2	0.9999	0.0008	0.0005	0.0173	0.9298	0.3380	0.2363	8.5938
3	0.9976	0.0557	0.0380	1.2634	0.9254	0.3483	0.2391	8.4735
4	0.9999	0.0006	0.0003	0.0140	0.9206	0.3594	0.2466	8.2557
5	0.9999	0.0006	0.0003	0.0128	0.9192	0.3626	0.2492	9.8812
6	0.9973	0.0591	0.0365	1.1601	0.9191	0.3629	0.2456	8.8101
7	0.9993	0.0291	0.0161	0.4872	0.9175	0.3665	0.2549	8.7113
8	0.9999	0.0007	0.0004	0.0135	0.9165	0.3687	0.2642	8.5379
9	0.9999	0.0007	0.0003	0.0136	0.9158	0.3701	0.2348	7.1926
10	0.9999	0.0007	0.0003	0.0134	0.9154	0.3711	0.2575	8.4557
11	0.9999	0.0008	0.0004	0.0132	0.9133	0.3755	0.2332	9.3724
12	0.9999	0.0007	0.0004	0.0145	0.9132	0.3758	0.2529	7.7932
13	0.9999	0.0006	0.0003	0.0136	0.9125	0.3773	0.2631	8.9485
14	0.9999	0.0011	0.0004	0.0160	0.9120	0.3785	0.2492	7.8751
15	0.9999	0.0006	0.0003	0.0120	0.9118	0.3788	0.2575	9.5561
16	0.9999	0.0023	0.0010	0.0318	0.9111	0.3803	0.2565	8.8653
17	0.9921	0.1014	0.0654	2.0231	0.9110	0.3807	0.2481	9.5777
18	0.9983	0.0462	0.0286	0.8913	0.9109	0.3809	0.2424	8.0028
19	0.9999	0.0006	0.0003	0.0129	0.9107	0.3812	0.2726	9.0693
20	0.9953	0.0779	0.0493	1.5312	0.9100	0.3827	0.2540	9.9345
21	0.9999	0.0008	0.0004	0.0156	0.9096	0.3836	0.2564	8.0093
22	0.9999	0.0007	0.0004	0.0147	0.9088	0.3852	0.2498	7.7160
23	0.9990	0.0357	0.0219	0.7002	0.9088	0.3853	0.2463	8.5231
24	0.9999	0.0006	0.0004	0.0143	0.9075	0.3880	0.2464	8.5146
25	0.9920	0.1017	0.0646	2.2360	0.9074	0.3883	0.2491	8.7692
26	0.9986	0.0424	0.0247	0.7973	0.9066	0.3898	0.2491	8.2295
27	0.9970	0.0624	0.0387	1.2523	0.9065	0.3900	0.2676	10.3347
28	0.9999	0.0006	0.0003	0.0127	0.9062	0.3907	0.2440	7.6425
29	0.9999	0.0006	0.0004	0.0143	0.9058	0.3916	0.2412	8.3865
30	0.9999	0.0010	0.0004	0.0145	0.9048	0.3936	0.2494	8.4057
31	0.9726	0.1888	0.1301	4.0971	0.9046	0.3940	0.2506	8.2791
32	0.9999	0.0006	0.0003	0.0110	0.9039	0.3956	0.2501	9.7085
33	0.9368	0.2870	0.2097	6.5183	0.9032	0.3969	0.2859	10.8543
34	0.9999	0.0007	0.0003	0.0118	0.9009	0.4017	0.2514	10.4232

Table S1: Summary of four-feature GPR model performance in the final evaluation.

F. Thermodynamic Consistency

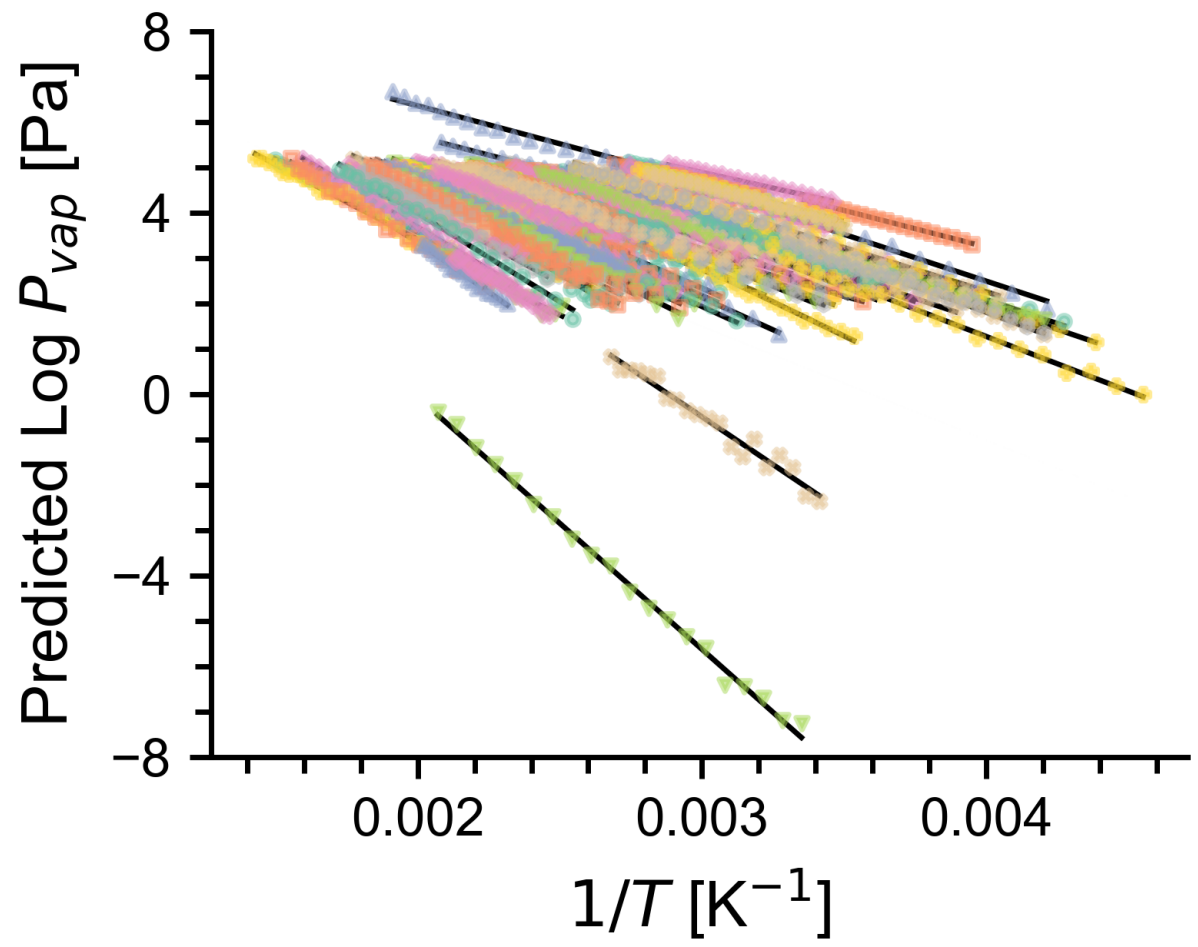

Figure S7: Predicted log vapor pressure vs inverse temperature for the holdout test group of the GPR dataset. Each color and marker combination represents a different molecule. The average R^2 and RMSE of fitted lines are 0.983 and 0.053 Pa, respectively.

G. ML Prediction Performance Comparison

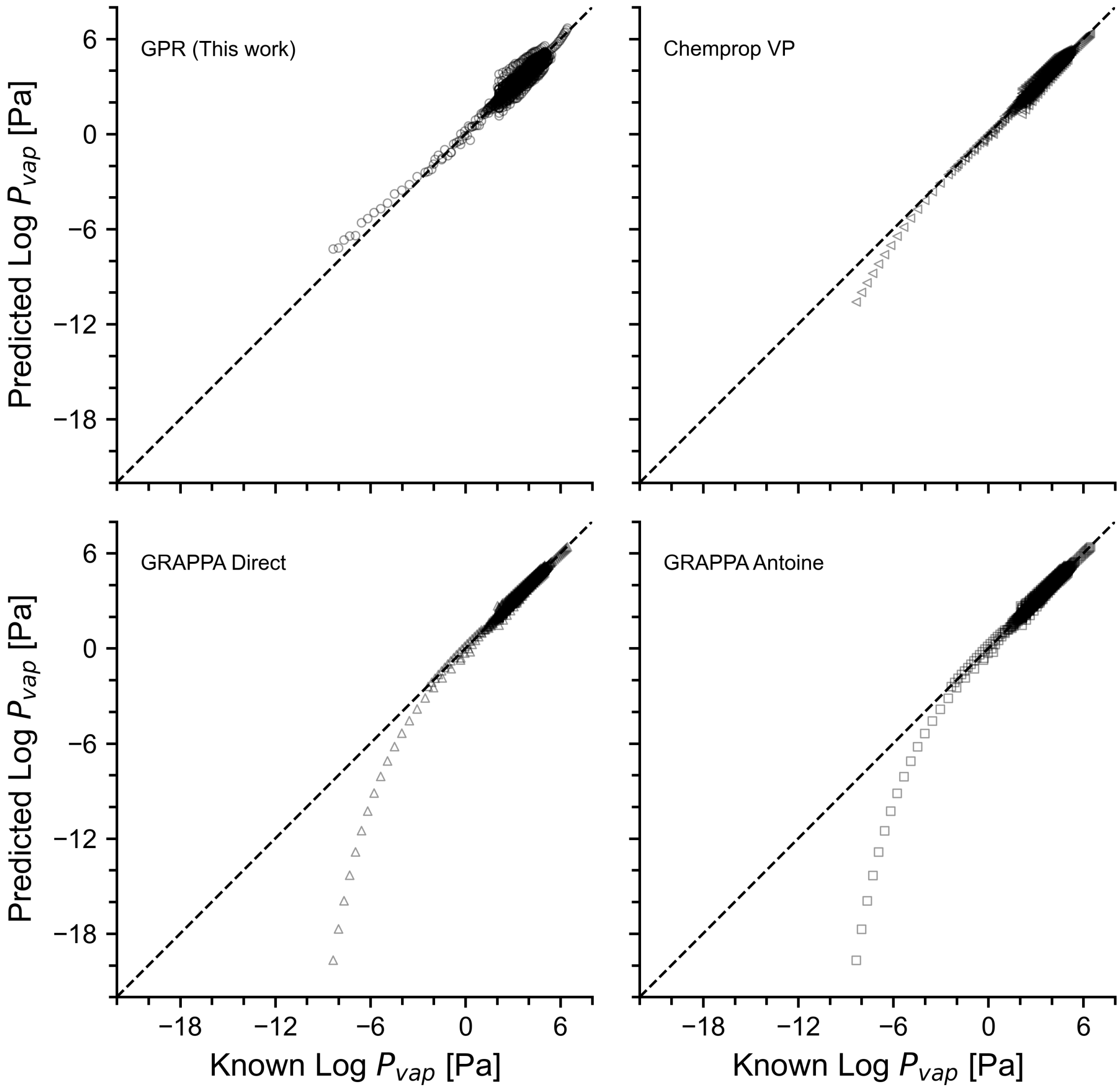

Figure S8: Predicted vs known vapor pressures for our GPR (top left), Chemprop VP[S13] (top right), GRAPPA Direct[S12] (bottom left) and GRAPPA Antione[S12] (bottom right) models. The RMSE for instances below 1 Pa is 0.5, 0.745, 3.631, and 3.631, for our GPR, Chemprop VP, GRAPPA Direct and GRAPPA Antione models, respectively. The R^2 for instances below 1 Pa is 0.964, 0.745, -0.908, and -0.908, for our GPR, Chemprop VP, GRAPPA Direct and GRAPPA Antione models, respectively.

REFERENCES

[S1] Nye Lubricants Inc., *NYE SYNTHETIC OIL 2001*.

[S2] W. R. Jones, Jr, M. J. Jansen, L. J. Gschwender, C. E. Snyder, Jr, S. K. Sharma, R. E. Predmore and M. J. Dube, *J. Synth. Lubr.*, 2004, **20**, 303–315.

[S3] C. G. Venier, W. R. Jones, Jr, M. J. Jansen and M. Marchetti, *Proceedings of the 10th European Space Mechanisms and Tribology Symposium, 24-26 September 2003, San Sebastián, Spain. Compiled by R. A. Harris. ESA SP-524, Noordwijk, Netherlands: ESA Publications Division*, 2003, **524**, 337–340.

[S4] C. G. Venier, E. W. Casserly, W. R. Jones, Jr, M. Marchetti, M. J. Jansen and R. E. Predmore, *Proceedings of the 36th Aerospace Mechanisms Symposium*, 2002.

[S5] Q. N. Nguyen and W. R. Jones, Jr, *Tribol. Trans.*, 2001, **44**, 671–677.

[S6] Nye Lubricants, *NYE SYNTHETIC OIL 1001*.

[S7] A. Rohatgi, *WebPlotDigitizer*.

[S8] W. L. Jorgensen and J. Tirado-Rives, *J. Comput. Chem.*, 2005, **26**, 1689–1700.

[S9] W. L. Jorgensen, D. S. Maxwell and J. Tirado-Rives, *J. Am. Chem. Soc.*, 1996, **118**, 11225–11236.

[S10] L. Martínez, R. Andrade, E. G. Birgin and J. M. Martínez, *J. Comput. Chem.*, 2009, **30**, 2157–2164.

[S11] A. P. Thompson, H. M. Aktulga, R. Berger, D. S. Bolintineanu, W. M. Brown, P. S. Crozier, P. J. in 't Veld, A. Kohlmeyer, S. G. Moore, T. D. Nguyen, R. Shan, M. J. Stevens, J. Tranchida, C. Trott and S. J. Plimpton, *Computer Physics Communications*, 2022, **271**, 108171.

[S12] M. Hoffmann, H. Hasse and F. Jirasek, *Chemical Engineering Journal Advances*, 2025, **22**, 100750.

[S13] Y.-H. Lin, H.-H. Liang, S.-T. Lin and Y.-P. Li, *Journal of the Taiwan Institute of Chemical Engineers*, 2024, 105926.
